# Identification of Cannabidiolic and Cannabigerolic Acids as MTDL AChE, BuChE, and BACE‐1 Inhibitors Against Alzheimer's Disease by In Silico, In Vitro, and In Vivo Studies

**DOI:** 10.1002/ptr.8369

**Published:** 2024-11-07

**Authors:** Rosa Maria Vitale, Andrea Maria Morace, Antonio D'Errico, Federica Ricciardi, Antimo Fusco, Serena Boccella, Francesca Guida, Rosarita Nasso, Sebastian Rading, Meliha Karsak, Diego Caprioglio, Fabio Arturo Iannotti, Rosaria Arcone, Livio Luongo, Mariorosario Masullo, Sabatino Maione, Pietro Amodeo

**Affiliations:** ^1^ Institute of Biomolecular Chemistry (ICB), National Research Council (CNR) Pozzuoli (NA) Italy; ^2^ Department of Experimental Medicine, Division of Pharmacology University of Campania "Luigi Vanvitelli" Naples Italy; ^3^ Department of Medical, Human Movement and Well‐Being Sciences University of Naples “Parthenope” Naples Italy; ^4^ Neuronal and Cellular Signal Transduction, Center for Molecular Neurobiology Hamburg (ZMNH) University Medical Center Hamburg‐Eppendorf (UKE) Hamburg Germany; ^5^ Institute of Human Genetics, University Medical Center Hamburg‐Eppendorf (UKE) Hamburg Germany; ^6^ Department of Pharmaceutical and Pharmacological Sciences University of Eastern Piedmont "A. Avogadro" Novara Italy

**Keywords:** alzheimer's disease, BACE‐1, beta‐amyloid peptide, cholinesterase enzymes, phytocannabinoids, molecular docking, molecular dynamics

## Abstract

Cannabidiolic (CBDA) and cannabigerolic (CBGA) acids are naturally occurring compounds from *Cannabis sativa* plant, previously identified by us as dual PPARα/γ agonists. Since the development of multitarget‐directed ligands (MTDL) represents a valuable strategy to alleviate and slow down the progression of multifactorial diseases, we evaluated the potential ability of CBDA and CBGA to also inhibit enzymes involved in the modulation of the cholinergic tone and/or β‐amyloid production. A multidisciplinary approach based on computational and biochemical studies was pursued on selected enzymes, followed by behavioral and electrophysiological experiments in an AD mouse model. The β‐arrestin assay on GPR109A and qPCR on TRPM7 were also carried out. CBDA and CBGA are effective on both acetyl‐ and butyryl‐cholinesterases (AChE/BuChE), as well as on β‐secretase‐1 (BACE‐1) enzymes in a low micromolar range, and they also prevent aggregation of β‐amyloid fibrils. Computational studies provided a rationale for the competitive (AChE) vs. noncompetitive (BuChE) inhibitory profile of the two ligands. The repeated treatment with CBDA and CBGA (10 mg/kg, i.p.) improved the cognitive deficit induced by the β‐amyloid peptide. A recovery of the long‐term potentiation in the hippocampus was observed, where the treatment with CBGA and CBDA also restored the physiological expression level of TRPM7, a receptor channel involved in neurodegenerative diseases. We also showed that these compounds do not stimulate GPR109A in β‐arrestin assay. Collectively, these data broaden the pharmacological profile of CBDA and CBGA and suggest their potential use as novel anti‐AD MTDLs.

## Graphical abstract

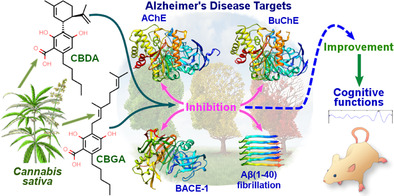

## 1 Introduction

Alzheimer's disease (AD) is a chronic neurodegenerative disease and the most common cause of dementia in the elderly population. AD is characterized by a progressive and irreversible cognitive decline that leads to memory loss, language and speech impairments, disorientation, and mood and personality changes, usually accompanied by psychosis, aggressivity, depression, and other symptoms of dementia that severely affect the quality of life of patients with AD (Koch and Spampinato 2022; Livingston et al. 2017). Although the etiology of AD is still unknown, genetics as well as environmental and metabolic risk factors, such as hypertension, diabetes, and hyperlipidemia, play a key role in the onset and progression of AD (Cheng et al. 2018; Elahi and Miller 2017). The hallmarks of AD include the formation of β‐amyloid plaques and neurofibrillary tau tangles and the severe deficit of the cholinergic system (Ferreira et al. 2021). In this view, two broad families of therapeutic approaches for AD involve the blocking of β‐amyloid peptide production, release or aggregation, or the promotion of its clearance, and the modulation of the pathways contributing to the cholinergic system.

Within this strategy, β secretase‐1 (BACE‐1) represents a well‐validated target to address AD since it is the main enzyme responsible for β‐amyloid peptide release (Prati et al. 2018).

PPARs, besides their better‐known role in metabolic diseases and inflammation, also represent interesting targets for approaches targeting β‐amyloid peptides. In fact, PPAR agonists have been shown to improve cognition and memory in experimental models of AD, ameliorating the disease‐related pathology (Wójtowicz et al. 2020). For example, PPARγ agonists have been shown to decrease Aβ accumulation, either by reducing Aβ production or enhancing its clearance, thus counteracting neuroinflammation and improving memory impairments both in several AD rodent models and in humans with mild‐to‐moderate AD (Escribano et al. 2010; Govindarajulu et al. 2018).

As for the cholinergic transmission, it ends with the hydrolysis of the neurotransmitter acetylcholine (ACh) by either acetylcholinesterase (AChE) or butyrylcholinesterase (BuChE), two serine hydrolase enzymes which represent well‐validated targets to alleviate AD symptoms. Under physiological conditions, BuChE plays a complementary role in the brain, accounting for about 10% of the ChE activity. However, BuChE contribution to ACh hydrolysis may become prominent under pathological conditions, as suggested by the combination of a decrease in AChE and stability or, even, an increase in BuChE levels observed in AD‐affected brains (Mushtaq et al. 2014). Moreover, due to its presence in senile plaques, BuChE has been recently associated with Aβ pathology (Darvesh et al. 2012) and both a reduction in Aβ plaque deposition and a higher resistance to cognitive impairment have been observed in BuChE knockout mice (Reid and Darvesh 2015). Altogether, these results support the role of the cholinergic system as a target for AD therapies, not only to alleviate symptoms but also to delay its progression. The activity of these enzymes can be regulated by competitive or noncompetitive inhibition, targeting their peripheral anionic site (PAS) and catalytic active site (CAS), located at the entrance and the bottom, respectively, of a narrow gorge which forms the ligand‐binding pocket (Bajda et al. 2013).

The intrinsic multifactorial nature of AD disease, and, in particular, the possibility of simultaneously targeting the two families of target described above, solicits an effort to search for or develop compounds behaving as multitarget‐directed ligands (MTDLs), that is, single molecules able to simultaneously modulate more than one molecular target involved in the pathology, thus maximizing the efficacy of the treatment and lowering the unwanted effects arising from the concomitant administration of different drugs (Rossi et al. 2021).

Natural compounds represent an invaluable reservoir of bioactive molecules, many of these traditionally used for ages as therapeutics to treat a wide range of diseases (Atanasov et al. 2021). Their high chemical and structural diversities, combined with the occurrence of specific functional groups able to engage polar interactions, render these molecules, particularly prone to interact with multiple‐related targets, resulting potentially effective in the treatment of multifactorial diseases. A representative example of natural compounds endowed with a multitarget profile is the meroterpenoid class of phytocannabinoids (pCBs) from the plant *Cannabis sativa* (Legare, Raup‐Konsavage, and Vrana 2022; Rathod and Agrawal 2024; Vitale, Iannotti, and Amodeo 2021). The most extensively studied pCBs are the so‐called “big‐4”, that is, cannabigerol (CBG), cannabichromene (CBC), cannabidiol (CBD), and Δ^9^‐tetrahydrocannabinol (Δ^9^‐THC), with the latter two being already approved drugs for the treatment of refractory epilepsy CBD and spasticity, nausea, and pain (CBD/Δ^9^‐THC) (Vitale, Iannotti, and Amodeo 2021). In particular, CBD has been reported to be effective in counteracting Δ^9^‐THC‐induced memory impairment (Castelli et al. 2023), to exert neuroprotective effects in different models of beta‐amyloid‐induced toxicity (Esposito et al. 2007; Karl, Garner, and Cheng 2017), and to decrease Aβ accumulation through PPARγ activation (Esposito et al. 2011).

Since, in our previous study, we identified cannabidiolic (CBDA) and cannabigerolic (CBGA) acids as dual PPARα/γ agonists (D'Aniello et al. 2019), we decided to evaluate the suitability of CBDA and CBGA to act as MTDLs against the well‐known enzymatic targets relevant in AD (AChE, BuChE, and BACE‐1 enzymes) (Maiuolo et al. 2023; Vitale et al. 2018) and to also extend the analysis to other emerging pathways related to neurological diseases (GPR109A and TRPM7) (Sun et al. 2015; Taing, Chen, and Weng 2023), to broaden the potential applications of these compounds in neurodegenerative disorders In fact, differently from their neutral counterpart, the pharmacological profile of acidic derivatives of pCBs still remains poorly investigated, especially in the context of neurological diseases.

To provide insights at different levels of biological complexity, we pursued a multidisciplinary approach, starting from *in silico* up to in vivo studies. Furthermore, computational studies, by elucidating and rationalizing the mechanism of action of bioactive compounds at the molecular level, could drive their optimization or the de novo design of nature‐inspired compounds.

## 2 Materials and Methods

### 2.1 Computational Methods

Starting ligands geometry were built with UCSF Chimera program (Pettersen et al. 2004), followed by initial energy minimization (EM) at the AM1 semiempirical level. The molecules were then fully optimized using the GAMESS program (Schmidt et al. 1993) at the Hartree–Fock level with the STO‐3G basis set and subjected to HF/6‐31G*/STO‐3G single‐point calculations to derive the partial atomic charges using the RESP procedure (Fox and Kollman 1998). Docking studies were performed with AUTODOCK 4.2 (Morris et al. 2009) following the protocol already published (Iannotti et al. 2020). The crystallographic structures of human AChE and BuChE (PDB: 7RB5 and 7BO3, respectively), two structures for human BACE‐1, representative of the flap open (PDB: 7MYI) or closed (PDB: 3TPP), along with the ligands, were processed with AutoDock Tools (ADT) package version 1.5.6rc1 (Morris et al. 2009) to merge nonpolar hydrogens and calculate Gasteiger charges. Grids for docking evaluation with a spacing of 0.375 Å and 70 × 70 × 70 points for AChE and BuChE and 70 × 70 × 60 points for BACE‐1, centered in the ligand‐binding pocket, were generated using the program AutoGrid 4.2 included in Autodock 4.2 distribution. Representative complexes for each ligand were completed by addition of all hydrogen atoms and underwent EM and then molecular dynamics (MD) simulations with pmemd.cuda module of Amber20 package (Case et al. 2020), using ff14SB force field for the protein and gaff2 parameters for the ligands. To perform MD simulations in solvent, the protein was confined in a TIP3P water periodic box exhibiting a minimum distance between solute atoms and box surfaces of 10 Å, using the tleap module of the AmberTools20 package. The systems were then neutralized by addition of counterions and subjected to EM and MD simulations following a previously published protocol (Iannotti et al. 2020). Production MD simulations were carried out at constant temperature (300 K) and pressure (1 atm) for 100–200 ns, with a time step of 2 fs. Cpptraj module of Amber20 package has been used to perform the analysis of MD trajectories, while UCSF Chimera 2004 has been used to draw the figures.

### 2.2 Purification of CBDA and CBGA

CBDA and CBGA were isolated from raw plant material according to the procedure of (Appendino et al. 2008). The strains of *Cannabis sativa* used for the isolation came from greenhouse cultivation at CRA‐CIN, Rovigo (Italy), where voucher specimens are kept for each of them, and were collected in July 2021. The purity of the compounds (> 99%) was confirmed by NMR analysis (Figures S1 and S2), and the structures were identified in accordance with the literature data.

### 2.3 Biochemical Assays

Acetylcholinesterase from *Electrophorus electricus* AChE, butirrylcholinesterase from equine serum BuChE, acetylthiocholine, butirrylthiocholine, 5′,5′‐dithiobis‐2‐nitrobenzoic acid (DTNB), thioflavine T, and the fluorescent peptide for β‐secretase activity were purchased from Sigma‐Aldrich (Milano, Italy). Human β‐amyloid peptide (1–40, cat. ab120479) was obtained from Abcam (Cambridge, UK). Mouse BACE‐1 was from Life Technology. HCA2‐Tango was a gift from Bryan Roth (Addgene plasmid # 66396; http://n2t.net/addgene:66396; RRID:Addgene 66,396). HTLA‐HEK293 cells were kindly provided by Dr. Richard Axel and were provided by Dr. Gilad Barnea (Barnea et al. 2008). BACE‐1 activity was determined by a fluorimetric method essentially as previously reported (Vitale et al. 2018). The reaction mixtures, prepared in 50 mM ammonium acetate buffer, pH 4.5 supplemented with 1 mM triton ×‐100, contained 2.1 ng/μL mouse BACE‐1, and different amounts of the inhibitors. After 10 min at room temperature, the reaction was initiated by adding 100 nM final concentration of the fluorescent peptide substrate. The increase in fluorescence was followed kinetically using excitation and emission wavelengths of 320 and 420 nm, respectively, using the Enspire Multimode Plate Reader (PerkinElmer). The rate was derived from the linear portion of the kinetics, usually in the first 30 min of the reaction. The concentration of inhibitor required to reduce the enzymatic activity to 50% (IC_50_) was derived from semi logarithmic plots. A*β*_1–40_ self‐aggregation inhibition assay was evaluated by incubating 96 μM peptide in 12 μL of 200 mM sodium phosphate buffer (pH 8.0) containing 0.5% (v/v) DMSO at 37°C for 24 h in the absence or presence of the inhibitors. Then, 0.5 mL of 1.6 μM thioflavine T in 50 mM glycine‐NaOH buffer (pH 8.5) was added, and the fluorescence intensity was measured over a 300 s time scan, using excitation and emission wavelengths of 446 and 490 nm, respectively (slits were set to 10 nm for both the excitation and the emission beams), by a Cary Eclipse Spectrofluorimeter (Agilent). The fluorescence values at plateau were averaged over a scan of at least 2 min. The extent of the inhibition was calculated from the decrease in the fluorescence signal after the subtraction of the background fluorescence. The concentration leading to 50% residual self‐aggregation (IC_50_) was derived from a semi logarithmic plot.

### 2.4 In Vivo Experimental Design

Soluble amyloid‐β (1–42) peptide (sAβ) was sourced from Tocris (Bristol, UK). Stock solutions were initially prepared by dissolving the peptide in DMSO to a concentration of 1 mM to address preaggregates, and then further diluted to 40 μM with double‐distilled water. These stock solutions were stored at −20°C. For injections, a fresh solution was prepared by diluting the stock with sterile double‐distilled water (vehicle) to achieve a final concentration of 4 μM. CBGA and CBDA were administered intraperitoneally at the dose of 10 mg/kg, or vehicle (DMSO diluted 1:9 in distilled water), from days 3 to 10 following the sAβ injection (H. Y. Kim et al. 2016; Mhillaj et al. 2018; Tucci et al. 2014).

A total number of 56 CD1 male mice (7–8 weeks), purchased from Envigo (Italy), were housed under controlled illumination and environmental conditions for 1 week before the commencement of experiments. The experimental procedures were approved by the Animal Ethics Committee of the University of Campania “Luigi Vanvitelli,” Naples. Animal care complied with the IASP and European Community (E.C. L358/1 18/12/86) guidelines on the use and protection of animals in experimental research (MOH project N. 627/2022‐PR). All efforts were made to minimize animal suffering and to reduce the number of animals employed. In this study, soluble amyloid‐β (1–42) peptide (sAβ) (5 μL) was slowly injected into the lateral ventricle of mice using a 10‐μL Hamilton microsyringe. The injection coordinates, based on the Paxinos and Franklin mouse brain atlas, were AP = −0.5 mm, ML = 1 mm, and DV = −2.3 mm. As a control, sham mice received an intracerebroventricular (i.c.v.) injection of an equal volume of vehicle solution. To prevent solution reflux along the needle track, the needle was left in place for an additional 5 min postinjection. AD‐like symptoms began to appear by the seventh day postinjection, as documented in previous studies (Mhillaj et al. 2018). To evaluate the effectiveness of CBGA and CBDA in mitigating or reversing sAβ‐induced AD‐like symptoms, we administered both drugs (CBGA and CBDA at 10 mg/kg i.p.) or a vehicle through repeated dosing regimens. The dose was based on previous (Anderson et al. 2019; Moore et al. 2023). Behavioral assessments were conducted 10 days after surgery. Electrophysiological studies were performed following the behavioral tests to confirm the presence of cognitive impairments. Mice were randomly assigned to one of four experimental groups for the repeated treatments: Sham/vehicle, sAβ/vehicle, sAβ/CBGA, and sAβ/CBDA. Each group was assigned an alphanumeric code, and all procedures were carried out by operators who were blinded to the group assignments.

### 2.5 Tail Suspension Test (TST)

The depressive‐like behavior was assessed using the TST. Mice were individually hung by their tails from a horizontal rod (50 cm above the floor) using adhesive tape placed about 2 cm from the end of the tail. The period of immobility, measured in seconds, was observed during the final 4 min of a 6‐min test by a timer. Immobility was defined as a lack of any escape‐related movements. Mice were considered immobile if they exhibited no body motion, hanging passively and entirely still.

### 2.6 Novel Object Recognition Test (NOR)

The NOR test, used to assess recognition memory, consists of three phases: Habituation, familiarization, and testing. During the habituation stage, conducted the day before the actual test, mice were allowed to freely explore a dimly lit polyvinyl chloride (PVC) box (40 × 30 × 30 cm) for 10 min, without any objects present, to familiarize themselves with the environment. During the familiarization phase, each mouse had 5 min to explore two identical objects placed in opposite corners at the back of the box. In the test phase, conducted 1 h after the familiarization session, one of the two identical objects was replaced with a novel, different object. The duration of time spent exploring each object was recorded. The results were expressed as a recognition index (NOR index), calculated using the following formula: (time spent exploring the novel object—time spent exploring the familiar object)/total exploration time (Guida et al. 2022). In acute therapeutic tests, the drug or placebo was administered 15 min before the familiarization trial.

### 2.7 Long‐Term Potentiation (LTP)

LTP recording was used to investigate memory and synaptic plasticity. Mice were first anesthetized with urethane (1.5 g/kg, i.p.) and fixed in a stereotaxic device (David Kopf Instruments, Tujunga, CA). Body temperature was maintained at 37°C with a temperature‐controlled heating pad (Harvard Apparatus Limited, Edenbridge, Kent). Extracellular field recording of LTP was performed 7 days after β‐amyloid injection. For LEC‐DG pathway recording, the skull was exposed, and a hole was drilled for the placement of a recording electrode into the DG (AP: −2.1 mm from bregma, L: 1.5 mm from midline; and V: 1.2 mm below dura) and a stimulating electrode into the LEC (AP: −4.0 mm from bregma; L: 4.5 mm from midline; and V: 2.9 mm below the dura). The stimulating and recording electrodes were lowered slowly into the LEC and DG, respectively, until a field excitatory postsynaptic potential (fEPSP) induced by test pulses (0.2 ms in duration delivered at the frequency of 0.033 Hz) was observed. After stabilization of the responses, a baseline was recorded for 30 min, and a high‐frequency stimulation (TBS, consisting of 6 trains, 6 bursts, and 6 pulses at 400 Hz; interburst interval: 200 ms; and intertrain interval: 20 s) was applied in the LEC to stimulate the perforant path fibers for inducing LTP.^13^ LTP was considered as an increase in the amplitude and slope of the fEPSPs that exceeded the baseline by 20% and lasted for at least 30 min from the TBS. After TBS, the recording of the fEPSPs was continued for 90–120 min. Field recordings were performed with a tungsten microelectrode (1–5 Mohm), and EPSPs were recorded at 20 kHz every 30 s for 60 min. In addition, the excitatory responses were amplified (×100), filtered at 5 kHz, and digitized by an interface (Digidata 1320A, Axon Instruments, Indonesia) connected to a computer on which the analysis software (WinLTP 2.10) was installed. At the end of experiments, mice were euthanized with lethal dose of urethane.

### 2.8 RNA Extraction and Quantitative PCR (qPCR)

Total RNA was isolated from the mouse hippocampus by use of RNeasy Micro Kit (cat# 74004, Quiagen IT) followed by spectrophotometric quantification. Subsequently, the RNA integrity number (RIN) for each sample was analyzed on the Agilent 2100 bioanalyzer. Purified RNA was then reverse‐transcribed by the use of the iScript cDNA Synthesis Kit (cat# 1708841 Biorad).

Quantitative PCR (qPCR) was carried out in a real‐time PCR system CFX384 (Bio‐Rad) using the SYBR Green PCR Kit (Cat# 1725274, Bio‐Rad for mRNAs). Each sample was amplified simultaneously in quadruplicate in a one‐assay run with a nontemplate control blank for each primer pair to control for contamination or primer‐dimer formation, and the cycle threshold (Ct) value for each experimental group was determined. The housekeeping genes ribosomal protein S16 were used to normalize the Ct values, using the 2^^−ΔCt^ formula.

The primer sequences used were murine TRPM7 forw 5’‐AGGATGTCAGATTTGTCAGCAAC‐3′; murine TRPM7 rev 5’‐CCTGGTTAAAGTGTTCACCCAA‐3′; murine S16 forw 5’‐CTGGAGCCTGTTTTGCTTCTG‐3′; and murine S16 rev 5’‐CTGGAGCCTGTTTTGCTTCTG‐3′.

### 2.9 β‐Arrestin Recruitment Assay

An adapted reporter gene method from the published protocol (Kroeze et al. 2015) was used with minor changes. HTLA‐HEK293 cells stably expressing β‐arrestin2‐TEV and tTA‐driven luciferase were kept in complete Dulbecco's modified Eagle's medium (DMEM) supplemented with 5% of fetal bovine serum, 5% of bovine calf serum, 2.5 μg/mL of puromycin, 50 μg/mL of hygromycin, 100 U/mL penicillin, and 100 μg/mL streptomycin at 37°C in a humidified incubator containing 5% CO_2_. Cells were transfected with the HCA2‐Tango plasmid (GPR109) using Lipofectamine and incubated overnight at 37°C. After 24 h, cells were transferred to a poly‐L‐Lysine coated 384‐well optical bottom plate and were stimulated in starving medium (DMEM supplemented with 1% fetal bovine serum and 1 x antibiotic/antimycotic (complete medium with 2.5 μg/mL of puromycin, 50 μg/mL of hygromycin, 100 U/mL penicillin, and 100 μg/mL streptomycin)) with the according substances (CBDA and CBGA) in 9 replicate wells for 20 h. The following day, a 20‐fold dilution of BrightGlo from Promega in assay buffer (20 mM HEPES, 1 × HBSS, pH 7.40) was added to the cells, and the luminescence was read using a Mithras^2^ LB 943 multimode microplate reader (Berthold technologies) with an integration time of 1 s/well. As a positive control, the cannabinoid receptor CNR2‐Tango plasmid was activated with its ligand JWH‐133 in duplicate replicates. Negative controls were the empty vector, and the receptors stimulated with vehicle solution (0.1% DMSO). The experiments were replicated 2–3 times with comparable results. The RLU values were depicted as mean ± SD values using GraphPad Prism Software Version 10.2.3.

### 2.10 Statistical Analysis

All results were plotted in graphs and analyzed statistically using GraphPad Prism 9.0 (GraphPad Software, USA). In all tests, *p* < 0.05 was considered significant. Data are presented as mean ± SEM. The D'Agostino‐Pearson normality test was used to assess the normal distribution of the data. The statistical analysis was performed using the Mann–Whitney's *U* test for two experimental groups or Kruskal–Wallis/Dunn's tests were used to analyze more than two experimental groups. Two‐way ANOVA followed by Holm‐Sidak's multiple comparison test was used to evaluate simultaneously the effect of two grouping variables (A and B) on a response variable.

## 3 Results

### 3.1 In Silico Study of CBDA and CBGA as Potential Inhibitors of AChE, BuChE, and BACE‐1 Enzymes

The potential ability of CBDA and CBGA to act as inhibitors of AChE, BuChE, and BACE‐1 enzymes was investigated *in silico* using a combined approach of molecular docking and MD simulations. The best poses in terms of cluster population and binding energy from molecular docking underwent 100 ns MD simulations to assess their stability. When a rearrangement occurred during the simulated period, MD was prolonged to allow further evolution of the system. Only the poses that resulted stable over a time‐window of 90 ns (see Figure S3) were considered representative and discussed in detail. Biochemical assays were then used to experimentally validate the computational results.

#### 3.1.1 CBDA/CBGA‐AChE Theoretical Complexes

**FIGURE 1:**
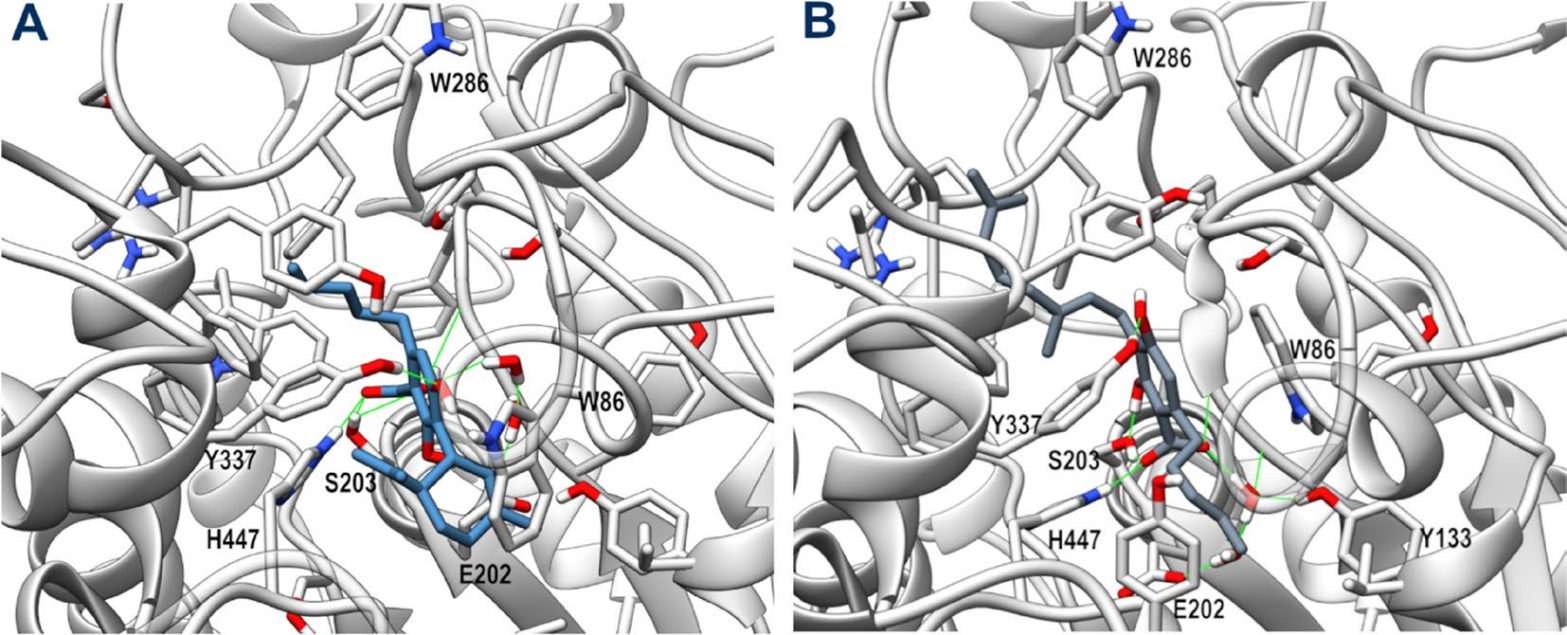
Representative MD frames of AChE (light gray) in complex with CBDA (steel blue, panel A) and CBGA (slate gray, panel B). A stick representation is used for heavy atoms of the ligand and protein sidechains within 5 Å of the ligand. Water molecules within 10 Å from the ligand involved in H‐bonds are shown in stick representation. Hydrogen, nitrogen, oxygen, and sulfur atoms are painted white, blue, red, and yellow, respectively. Half‐transparency is employed for the ribbon representation of protein regions overlying the ligand in the selected view. A green wire representation is adopted for H‐bonds.

The CBDA and CBGA starting docking poses only underwent moderate rearrangement during MD, mainly due to a translation toward the active site, driven by the polar interaction established with residues belonging to the CAS. As shown in Figure 1, the two ligands adopt an opposite orientation, with the terpenoid chain of CBGA extending toward the entrance of the gorge and forming hydrophobic interactions with Phe295, Phe297, and Val293, while the correspondent terpenoid ring of CBDA forms hydrophobic interactions with Trp86, His447, and Tyr337. However, the dihydroxy‐benzoic acid moiety of the two ligands engages a similar network of stable H‐bonds, involving its carboxylate group and both the catalytic Ser203 and His447 sidechains and the amide backbone of the residues forming the oxyanion hole, that is, Gly121 and Gly122. The 2,4 hydroxy groups of CBGA engage additional H‐bonds with Ser203 and Tyr337, while those of CBDA form a direct H‐bond with Tyr337 sidechain and a water‐mediated H‐bond with the Glu202 sidechain. A water‐mediated H‐bond is also observed between the carboxylate group of CBGA and both Glu202 and Tyr133 sidechains. The aromatic ring in both complexes forms π‐π interactions with Trp86. The overall arrangement of both pCBs into the active site of AChE and their direct interactions with residues of the catalytic site suggest that both ligands could act as AChE competitive inhibitors.

#### 3.1.2 CBDA/CBGA‐BuChE Theoretical Complexes

**FIGURE 2:**
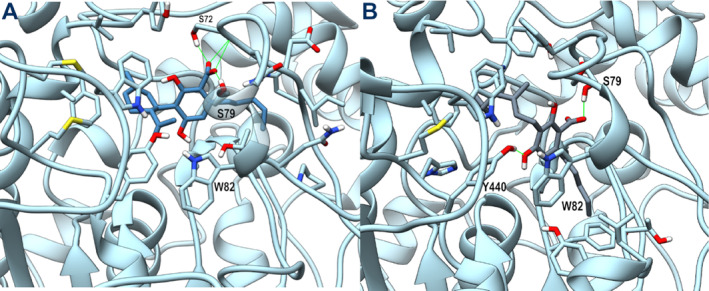
Representative MD frames of BuChE (light blue) in complex with CBDA (steel blue, panel A) and CBGA (slate gray, panel B). A stick representation is used for heavy atoms of the ligand and for protein sidechains within 5 Å of the ligand. Water molecules within 10 Å from the ligand involved in H‐bonds are shown in stick representation. Hydrogen, nitrogen, oxygen, and sulfur atoms are painted white, blue, red, and yellow, respectively. Half‐transparency is employed for the ribbon representation of protein regions overlying the ligand in the selected view. A green wire representation is adopted for H‐bonds.

The starting docking poses for both ligands were similar to those obtained for AChE. While MD of AChE complexes only resulted in a slight rearrangement finalized to reinforce the network of interactions with the catalytic site, MD of BuChE complexes produced a progressive loss of the polar interactions engaged with the catalytic residues, with a consequent drift from the CAS toward the PAS. To evaluate the stability of these new poses, MD was further prolonged by 100 ns (see plot in Figure S3). The representative poses for both ligands are shown in Figure 2: The terpenoid ring of CBDA is hosted in an aromatic cleft formed by Phe329, Tyr440, and Trp430, while the carboxylate group forms a bidentate interaction with Ser72 backbone and sidechain and Ser79 sidechain. CBGA adopts a similar orientation, with its terpenoid chain interacting with Phe329, Tyr332, and Trp430. However, its aromatic ring is more deeply inserted into the catalytic site, forming a π‐π stacking with Trp82 and an H‐bond between 4‐hydroxy group and Tyr440 sidechain. The different binding mode of both ligands toward the two cholinesterase enzymes could be ascribed to the larger catalytic site of BuChE respect to AChE, mainly due to the replacement of AChE Tyr337 with an Ala residue in BuChE. However, since the resulting binding modes of both ligands after MD are stable and located into the PAS of BuChE, it is possible to speculate that they could act as noncompetitive inhibitors of this enzyme.

### 3.2 Effect of CBDA and CBGA on Cholinesterase Activity

To test the inhibition capacity of CBDA and CBGA on cholinesterase activity, the kinetics parameters of AChE or BuChE were determined either in the absence or in the presence of different concentrations of the compounds. This procedure, although appropriate to determine the inhibition constant of the compounds, is also useful to assess the corresponding inhibition type. The data are reported in Figures S3 and S4 for representative experiments and collected in Tables S1 and S2 for all the experiments carried out. They indicate that both compounds act as competitive inhibitors of AChE and noncompetitive inhibitors of BuChE, respectively. In fact, regardless of the method applied for the calculation, the compounds induced, at all the sampled concentrations, an increase in *K*_m_, without affecting *V*_max_ values in the case of AChE (Figure S4, Table S1), and a decrease of *V*_max_ without affecting *K*_m_ in the case of BuChE (Figure S5, Table S2). The calculated *K*_i_ is 7.9 ± 4.3 μM (*n* = 6) for CBDA and 10.5 ± 2.9 μM (*n* = 7) for CBGA in the case of AChE and 6.7 ± 0.6 μM (*n* = 4) for CBDA and 23.3 ± 12.0 μM (*n* = 4) for CBGA in the case of BuChE. Thus, CBDA is slightly more efficient than CBGA in inhibiting AChE and significantly more potent on BuChE.

### 3.3 CBDA/CBGA‐BACE‐1 Theoretical Complexes

**FIGURE 3:**
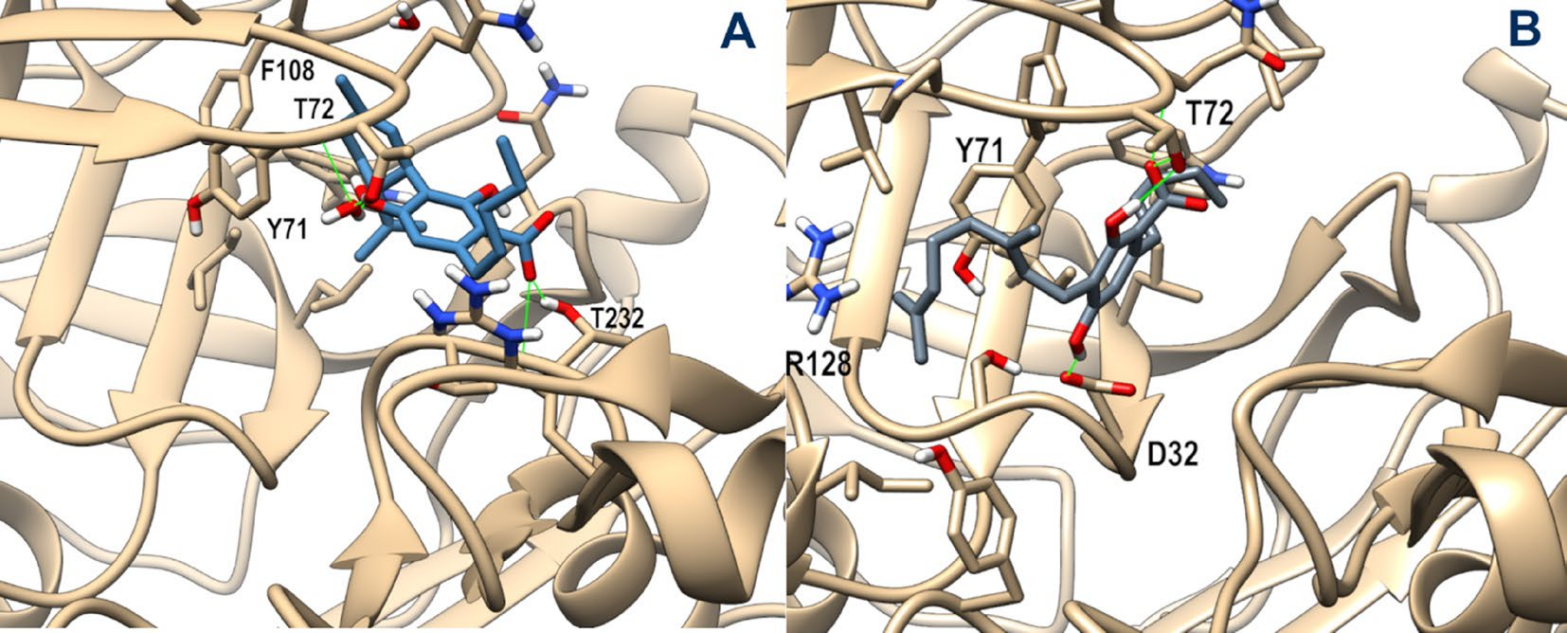
Representative MD frames of BACE‐1 (tan) in complex with CBDA (steel blue, panel A) and CBGA (slate gray, panel B). A stick representation is used for heavy atoms of the ligand and protein sidechains within 5 Å of the ligand. Water molecules within 10 Å from the ligand involved in H‐bonds are shown in stick representation. Hydrogen, nitrogen, oxygen, and sulfur atoms are painted white, blue, red, and yellow, respectively. Half‐transparency is employed for the ribbon representation of protein regions overlying the ligand in the selected view. A green wire representation is adopted for H‐bonds.

Then, we also explored the suitability of these compounds as beta‐secretase 1 (BACE‐1) inhibitors, using the same computational protocol described above. To account for the open and closed conformations of the flap loop that covers the BACE‐1 catalytic site, two different protein x‐ray structures (PDB id: 7MYI and 3TPP for open and closed conformations, respectively) were used as targets. However, due to the higher stability of the ligand docking poses obtained for the closed conformation of the flap loop during 100 ns MD, only these latter are discussed in detail. The representative MD frames for both ligand complexes are shown in Figure 3. During MD, the CBGA starting docking pose optimized both its π‐stacking with Tyr71 and a network of H‐bonds. In particular, the ligand carboxylate is H‐bonded to the backbone and sidechain of Thr72, and the 2‐hydroxy group interacts with the same sidechain, while the 4‐hydroxy group forms a stable H‐bond with the catalytic Asp32. The CBDA starting docking pose underwent a slight rearrangement during MD, then remaining stable over an additional 50 ns run. The terpenoid ring stacks against Tyr71 and forms hydrophobic interaction with Phe108, while the carboxylate group engages an H‐bond with Thr232 sidechain, and the 4‐hydroxy group forms a water‐mediated H‐bond with Thr72 sidechain.

### 3.4 Effect of CBDA and CBGA on BACE‐1 Activity

**FIGURE 4:**
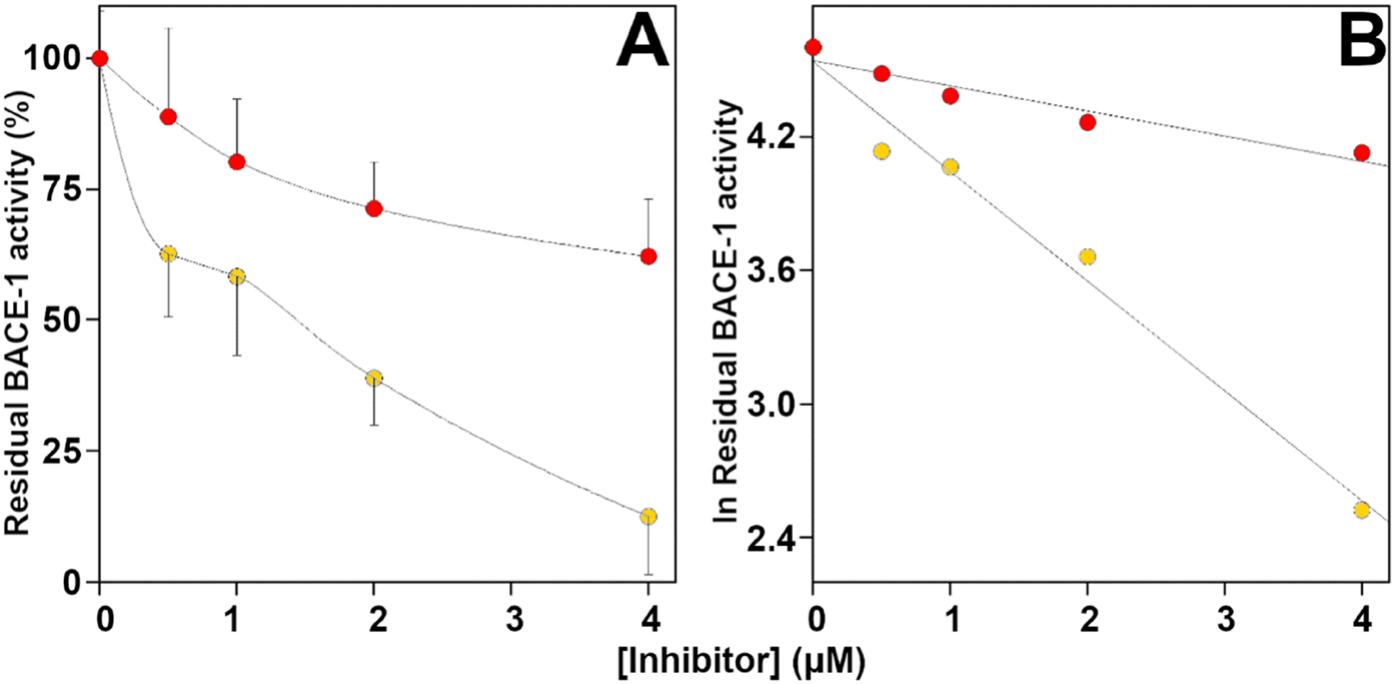
Effect of CBGA, and CBDA on BACE‐1 activity. Panel A. The residual BACE‐1 activity was determined in the absence or in the presence of the indicated concentration of CBDA (red circles) or CBGA (yellow circles) as reported in the Materials and Methods Section. The data of at least three different determinations were reported as mean percentage of that measured in the absence of the inhibitor. Panel B. Logarithmic transformation of the data shown in panel A.

The effect of CBDA and CBGA on BACE‐1 activity was checked using a fluorometric method in which the hydrolytic activity of mouse BACE‐1 is revealed by the formation of a fluorescence product obtained from a mutated and labeled Aβ peptide. The results reported in Figure 4A clearly show that CBGA and CBDA both inhibit BACE‐1 activity in a dose–response manner. The data treated in a semilogarithmic way (Figure 4B) allow the calculation of the IC_50_, and the values obtained, 1.4 ± 0.1 μM for CBGA and 6.1 ± 0.2 μM for CBDA, indicate that the former is roughly 4 times more efficient in inhibiting mBACE‐1.

### 3.5 Effect of CBDA and CBGA on Amyloidogenesis

**FIGURE 5:**
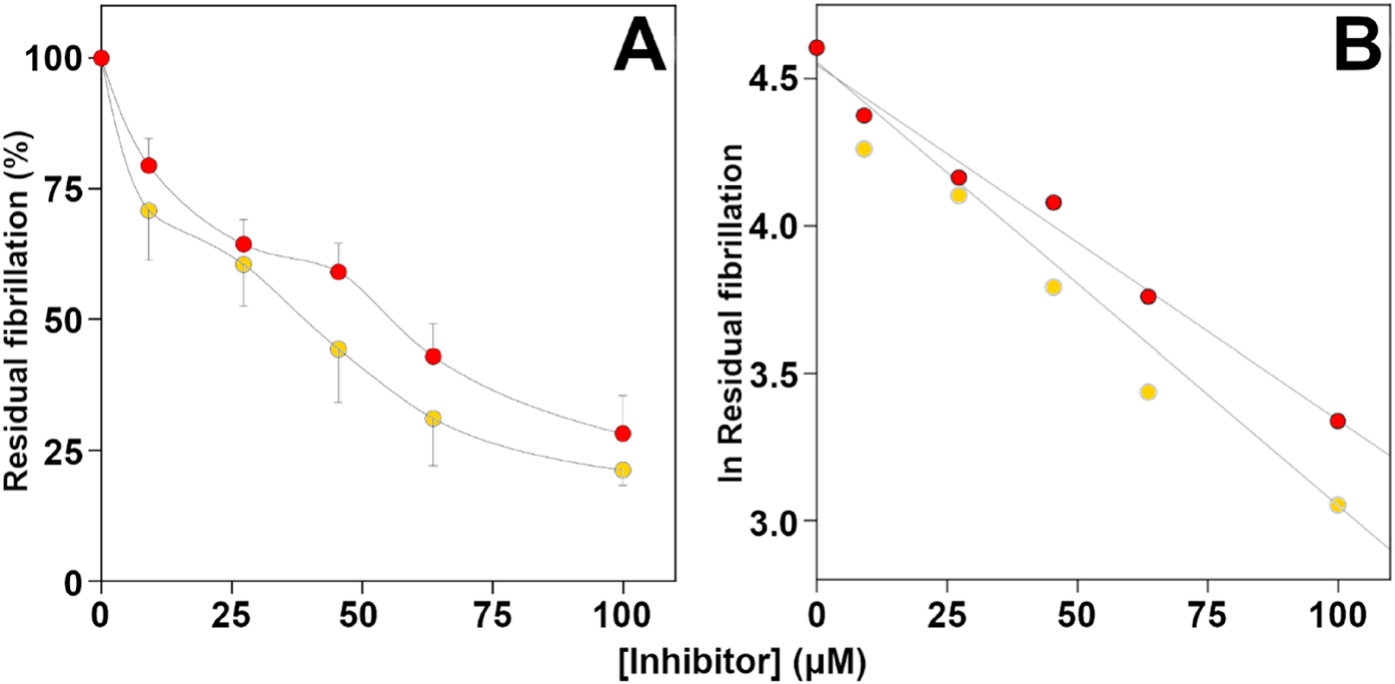
Effect of CBGA and CBDA on amyloidogenesis. Panel A: The amount of Aβ 1–40 self‐aggregation was determined in the absence or in the presence of the indicated concentration of CBDA (red circles) or CBGA (yellow circles) as reported in the Materials and Methods Section. The data of at least three different determinations were reported as a mean percentage of that measured in the absence of the inhibitor. Panel B. Logarithmic transformation of the data shown in panel A.

Encouraged by the results described above, next, we tested the potential efficacy of CBDA and CBGA in preventing β‐amyloid fibril formation. The data shown in Figure 5A indicate that both compounds inhibit the fibrillation of the Aβ 1–40 peptide to the same extent. However, although comparable IC_50_ values (47.7 ± 2.1 μM for CBGA vs. 57.5 ± 0.8 μM for CBDA) can be calculated from the semilogarithmic plot of the data reported in Figure 5B, a slightly higher efficiency was found for CBGA.

### 3.6 CBGA and CBDA Do Not Activate GPR109A in β‐Arrestin Assay

GPR109A (also known as acid receptor 2 (HCAR2)) plays a critical role in neuroinflammation related to neurological diseases (Taing, Chen, and Weng 2023). Since preliminary data on KO mice for GPR109 suggested a possible involvement of this receptor in mediating the anti‐inflammatory effect of CBDA (unpublished results), we decided to evaluate this receptor as a direct target for the investigated compounds. Therefore, we applied the β‐arrestin assay for GPR109A and stimulated HTLA‐HEK 293 cells expressing HCA2 receptors with CBDA and CBGA in different doses. The measured luminescence revealed similar values for cells stimulated with the substances in comparison to the negative control (vehicle solution) indicating no recruitment of β‐arrestin and no direct receptor activation (Figure S6).

### 3.7 Evaluation of CBDA and CBGA on Cognitive Impairment in an AD Mouse Model

**FIGURE 6:**
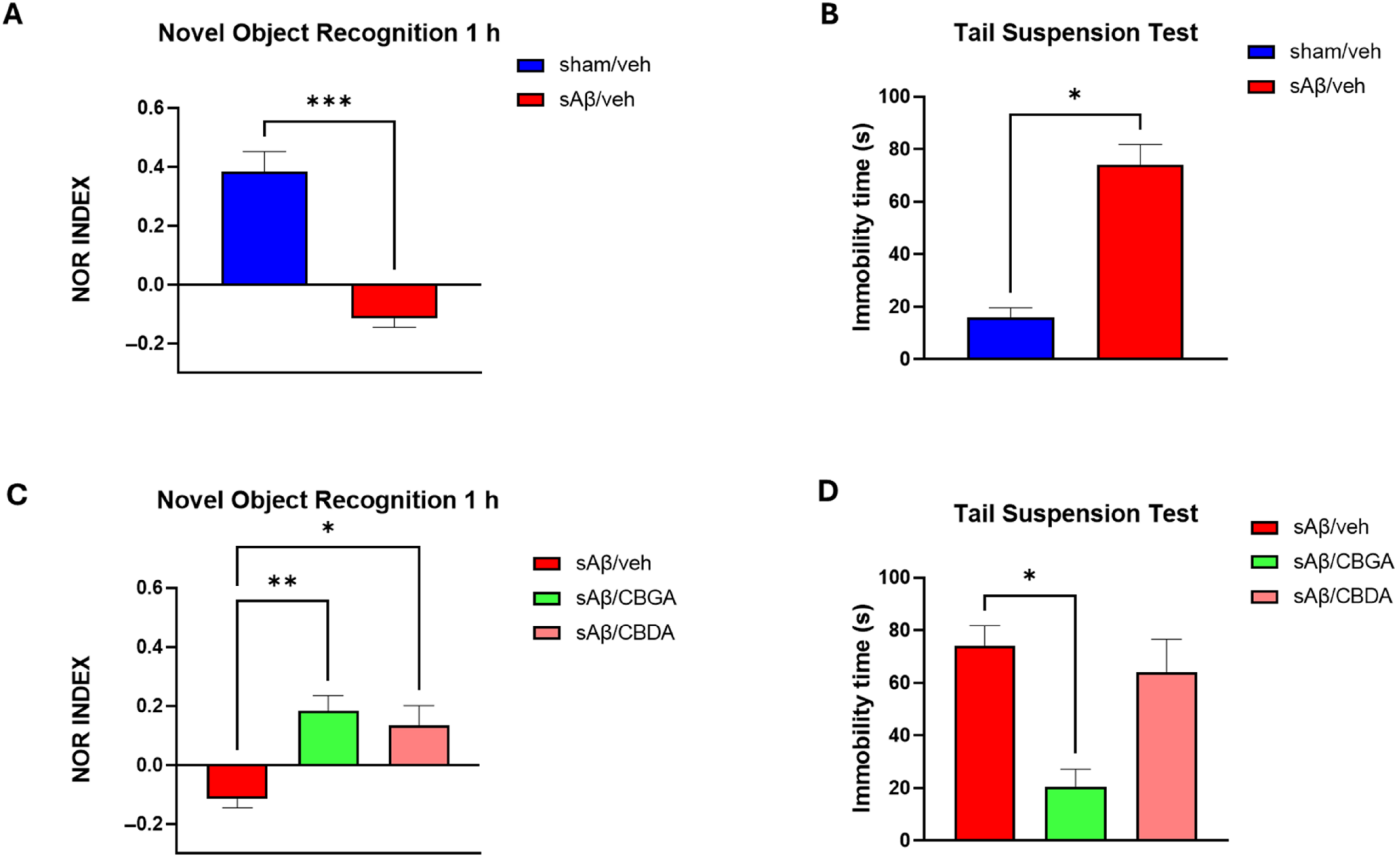
Panel A: Recognition index in Aβ‐injected mice versus control mice, at 10 days postinjection in novel object recognition test. Panel B: Immobility time in Aβ‐injected mice versus control mice, at 10 days postinjection in tail suspension test. Kruskal–Wallis/Dunn's tests were used to analyze more than two experimental groups. Panel C: Effect of repeated treatment with vehicle, CBGA, and CBDA (10 mg/kg i.p.) on discrimination ability, indicated as the NOR index in the novel object recognition test at 1 h, in a model of AD induced by sAβ injection‐like behavior in the tail suspension test, in a model of AD induced by sAβ injection. The statistical analysis was performed using the Mann–Whitney's *U* test for two experimental groups. Each bar represents the mean, and the vertical lines indicate SEM for 4–7 mice/group.

The efficacy of the repeated treatment of investigated compounds (10 mg/kg, i.p.) was evaluated using behavioral assays such as the NOR test and TST for cognitive performance and depressive‐like behavior, respectively. Similarly to our previous findings (Infantino et al. 2022), on the 10th‐day postinduction, the administration of soluble amyloid β peptide 1–42 (sAβ) caused a significant reduction in discrimination ability in mice in the sAβ/veh group compared to the control group (−0.115 ± 0.030 vs. 0.383 ± 0.069, *p* = 0.028) (Figure 6A), and immobility recorded during the test was significantly higher in AD mice compared to the Ctrl group (74.250 ± 7.465 vs. 15.750 ± 3.816, *p* = 0.00006) (Figure 6B).

Repeated treatment with CBGA improved cognitive performance in mice in the sAβ/CBGA group compared to animals treated with sAβ/veh (0.183 ± 0.052 vs. −0.115 ± 0.030, *p* = 0,0038). Similarly, the use of CBDA enhanced the discrimination abilities of mice in the sAβ/CBDA group (0.133 ± 0.068 vs. −0.115 ± 0.030, *p* = 0.013) (Figure 6C).

CBGA administration was effective in improving depressive‐like behavior in the sAβ/CBGA group compared to the vehicle‐treated group (20.500 ± 6.764 vs. 74.250 ± 7.465 *p* = 0.028). In contrast, CBDA administration was not significantly effective in reducing immobility times in the test (64.000 ± 12.523 vs. 74.250 ± 7.465, *p* > 0.99) (Figure 6D).

#### 3.7.1 Repeated Administration of CBGA and CBDA Reverses sAβ‐Induced LTP Impairment in the Hippocampus

**FIGURE 7:**
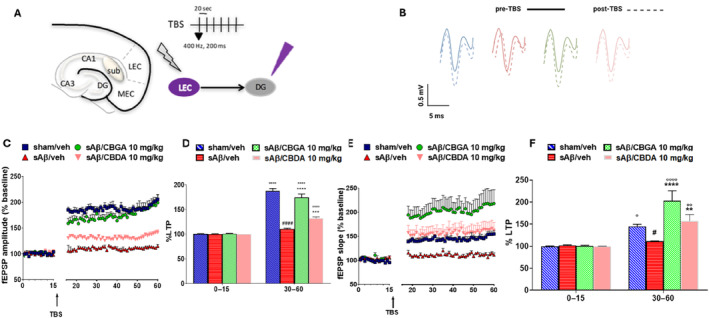
In vivo LTP recordings in the LEC‐DG pathway. A. Schematic representation of the stimulation electrode placement (lateral entorhinal cortex, LEC) and recording electrode placement (dentate gyrus, DG). The protocol for theta burst stimulation (TBS) to evoke long‐term potentiation (LTP) in the DG consists of 6 trains, each with 6 bursts, each burst containing 6 pulses at 400 Hz, with an interburst interval of 200 ms and an intertrain interval of 20 s. B. Representation of individual fEPSP recorded in the DG before and after the TBS protocol. C. Graph showing the changes over time in fEPSP amplitude after the TBS protocol. Data are expressed as a percentage of amplitude relative to baseline (0–15 min). D. Bar graph of amplitude 30–60 min after TBS, normalized to baseline (mean ± SEM). Data were analyzed by two‐way ANOVA followed by Holm‐Sidak's multiple comparison test. Statistical significance was set at *p* < 0.05. Significant differences relative to baseline (0–15 min); #Significant differences compared to sham/vehicle; *Significant differences compared to sAβ/vehicle. E. Data representing the mean slope (mean ± SEM) after TBS induction, normalized to baseline values (0–15 min). F. Bar graph of slope data 30–60 min after TBS, normalized to baseline (mean ± SEM). Data were analyzed by two‐way ANOVA followed by Holm‐Sidak's multiple comparison test, with statistical significance set at *p* < 0.05. Each bar represents the mean, and the vertical lines indicate SEM for 4 mice/group.

To assess the effect of sAβ on long‐term synaptic plasticity in the hippocampus, the lateral entorhinal cortex‐dentate gyrus (LEC‐DG) pathway, which is involved in learning and memory processes, was analyzed as illustrated in Figure 7. As expected, the LTP evoked in the dentate gyrus of sham mice treated with vehicle induced a significant increase in amplitude (30–60 min: 187.4 ± 5.1% vs. 0–15 min: 100.96 ± 1.37%, *p* < 0.0001) and slope (144.93 ± 4.93% vs. 0–15 min: 99.33 ± 1.38%, *p* = 0.0116), as shown in Figure 7D–F. In contrast, in mice injected with sAβ and treated with vehicle, TBS in the LEC did not alter the fEPSP amplitude (30–60 min: 110.5 ± 1.73% vs. 0–15 min: 100 ± 0.8%, *p* = 0.5249; Figure 7C) and only marginally affected the slope (110.92 ± 0.86% vs. 101.9 ± 1.18%, *p* = 0.0204; Figure 7E), confirming that Aβ oligomers impair memory. Repeated treatment with CBGA significantly improved synaptic efficiency after LTP induction in terms of amplitude (30–60 min: 174.34 ± 6.97% vs. 0–15 min: 100.8 ± 0.61%, *p* < 0.0001; Figure 7D) and slope (203.21 ± 22.55% vs. 0–15 min: 100.94 ± 1.15%, *p* < 0.0001; Figure 7F). Similarly, repeated treatment with CBDA significantly increased the amplitude (30–60 min: 132.08 ± 2.83% vs. 0–15 min: 99.97 ± 0.024%, *p* = 0.0001; Figure 7D) and slope (157.46 ± 14.5% vs. 0–15 min: 99.95 ± 0.27%, *p* = 0.0036; Figure 7F). Finally, a two‐way ANOVA with repeated measures identified significant effects for treatment (F (3, 12) = 57.92, *p* < 0.0001), time (F (1, 12) = 495.2, *p* < 0.0001), and treatment x time interaction (F (3, 12) = 60.73, *p* < 0.0001) for amplitude. Similarly, significant effects for treatment (F (3, 12) = 7.592, *p* = 0.0042), time (F (1, 12) = 61.80, *p* < 0.0001), and treatment × time interaction (F (3, 12) = 7.975, *p* = 0.0034) were also observed for slope.

### 3.8 Repeated Administration of CBGA and CBGA Restores the Physiological Expression Level of *Trpm7*

**FIGURE 8:**
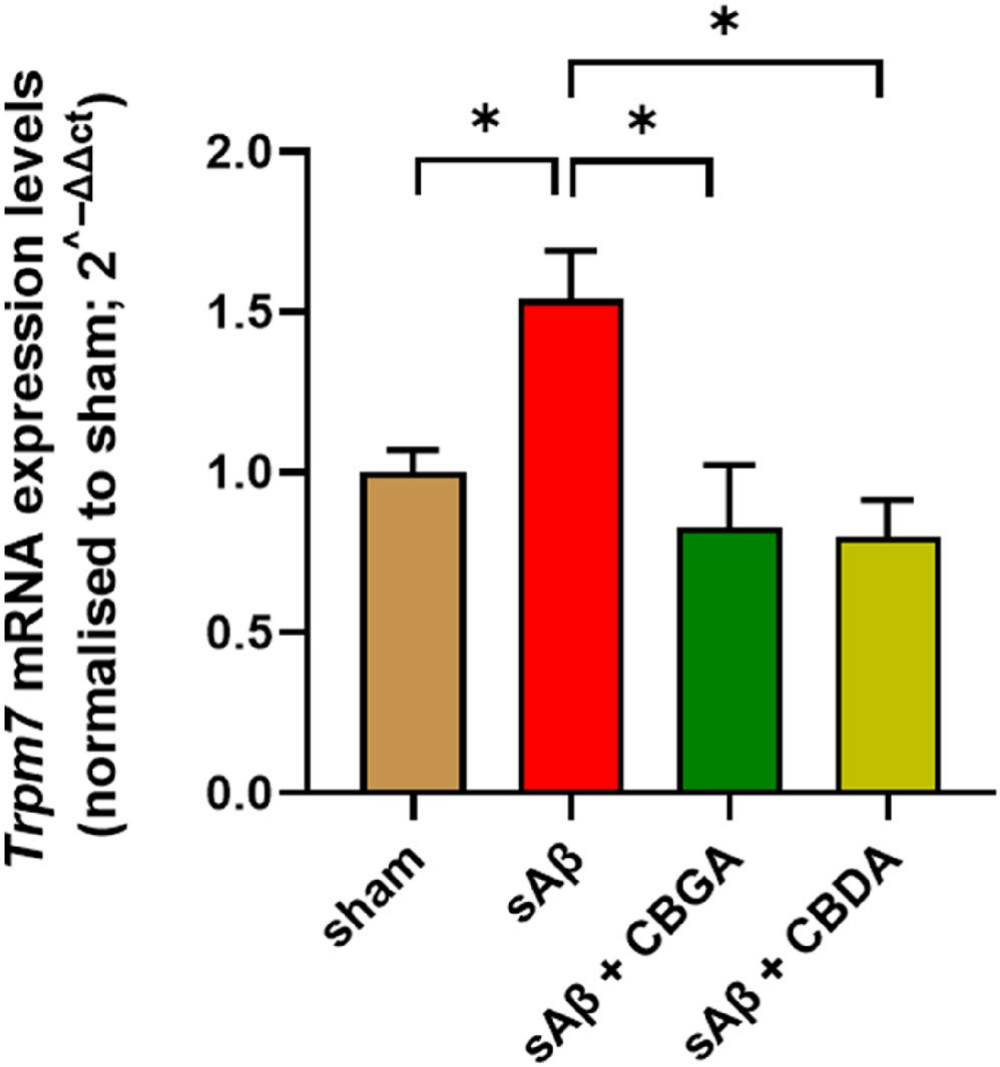
Effect of β‐amyloid peptide and CBDA and CBGA treatment on TRPM7 expression levels in the hippocampus. *p < 0.05 vs. the indicated groups calculated by ANOVA.

*Trpm7* gene encodes for the transient receptor potential melastatin 7 (TRPM7), a receptor channel involved in the homeostasis of Ca^2+^ and Mg^2+^ and highly expressed in neuronal cells. Due to the emerging role of TRPM7 in neurodegenerative diseases (Sun et al. 2015) and the recent findings of the inhibitory effect of negatively charged pCBs, in particular CBGA, on TRPM7 (Suzuki et al. 2023), we decided to investigate whether the treatment with CBGA and CBDA could also affect the expression level of *Trpm7* mRNA in the hippocampus. As shown in Figure 8, the *Trpm7* expression, measured by RT‐PCR, is significantly upregulated in treated mice with sAβ, a trend reverted to the basal level upon treatment (from day 3 to 10 postinduction) with both compounds.

## 4 Discussion

The aim of the study was to evaluate the suitability of CBDA and CBGA to act as MDTLs in the context of neurogenerative diseases, encouraged by our previous results on their activity as dual PPARα/γ agonists (D'Aniello et al. 2019) and by recent brain pharmacokinetic profile studies on acidic pCBs and in particular on CBDA, which showed that it can cross the blood–brain barrier (BBB), especially when administrated in a tween‐based vehicle (Anderson et al. 2019). The same study demonstrated a higher potency of CBDA as an anticonvulsant agent than CBD (Anderson et al. 2019). Interestingly, neither CBD nor CBG were detected in the brain following i.p. injection of CBDA and CBGA, respectively (Anderson et al. 2019), supporting the notion that the activity of these compounds is not due to their neutral counterparts.

As for other complex and multifactorial diseases, the use of MDTLs as a pharmacological strategy to slow down AD cognitive impairment is by far preferable over selective single‐target ligands, due to their ability to overcome the resistance to the treatment arising from the occurrence of redundant pathways (Rossi et al. 2021). For example, while under physiological conditions, BuChE only accounts for about 10% of the ChE activity, the dramatic increase in the BuChE/AChE ratio in AD‐affected brains makes its role predominant in Ach hydrolysis (Mushtaq et al. 2014).

In this study, we pursued a combined computational and experimental approach to evaluate the propensity of CBDA and CBGA to act as MTDL agents against molecular targets relevant to AD, such as AChE, BuChE, and BACE‐1 enzymes. Although in principle counterintuitive, being the classical inhibitors of these enzymes endowed with a positive charge to better mimic the natural substrate, as in the case of cholinesterases, or to interact with negatively charged aspartate residues in the case of BACE enzyme, few examples have been reported for negatively charged inhibitors (Tommonaro et al. 2016). In particular, the occurrence of shared structural features, such as the terpenoid chain and an aromatic scaffold, between the ChEI avarol derivatives and the investigated compounds, prompted us to investigate *in silico* the potential ability of CBDA and CBGA to act as inhibitors of these enzymes.

We used a computational protocol based on molecular docking and MD simulations on a 100 ns time scale to ensure a deeper exploration of the conformational space accessible to the ligands and allow possible rearrangements from the starting docking poses. In this way, it was possible to predict a different behavior for both the investigated compounds in the inhibition profile between the two close homolog cholinesterase enzymes, then confirmed by biochemical assays. In fact, both ligands behave as competitive inhibitors toward AChE and noncompetitive inhibitors toward BuChE. The different activity profile arises from the different binding modes adopted by the ligands in the active site of the enzymes: in the AChE complexes, both ligands engage polar interactions in the CAS, namely with catalytic residues Ser203 and His447, while in BuChE complexes, this binding mode was unstable during MD, probably because of its wider active site, resulting from the substitution of the bulky AChE Tyr337 with an alanine residue in BuChE. Instead, both ligands stably interact with a more external region in BuChE involving residues in the PAS. Thus, the network of polar interactions directly engaged by the carboxylate group, as well as the occurrence of water bridges between the carboxylate groups and protein residues in the complexes with cholinesterase enzymes, appears to allow a favorable accommodation of this group in the active site. Then, the potential activity of BACE‐1 for these compounds was investigated. The computational data suggested that both ligands could stably bind close to the catalytic site of this enzyme too, with CBGA forming a direct H‐bond with the catalytic Asp32 through the 4‐hydroxy group. A direct interaction with the catalytic aspartate residues was not observed with CBDA, probably due to its more rigid scaffold, which, however, stably interacts with the aromatic residues of the flap loop.

The biochemical assays confirmed the ability of these compounds to inhibit BACE‐1 in a low micromolar range. Moreover, both compounds are also able to directly counteract β‐amyloid self‐aggregation by inhibiting the fibrillation of the Aβ 1–40 peptide with the same potency, showing both a direct and a BACE‐1‐mediated effect in counteracting β‐amyloid formation. Spurred by preliminary observations in KO mice (data not shown), we also evaluated GPR109A, a GPCR involved in neuroinflammation, as a potential target. However, both compounds were unable to activate this receptor in the β‐arrestin recruitment assay, although further studies are required in this direction since the occurrence of a β‐arrestin–independent activation via biased signaling cannot be ruled out. Once assessed the activity on these targets, we decided to also evaluate their in vivo efficacy in contrasting cognitive impairment, using behavioral tests conducted on AD mice models. In fact, both their actions as dual PPAR agonists and as inhibitors of the aforementioned cholinesterase enzymes have been proven to counteract cognitive impairment in AD animal models (Aksoz, Akyol, and Korkut 2024; Sato et al. 2011; Wójtowicz et al. 2020). Indeed, the treatment with both compounds revealed an enhancement in cognition (discriminative memory) in βA‐mice treated with both compounds (10 mg/kg, i.p.). The efficacy of the compounds following i.p. administration also demonstrate that both compounds are able to cross the BBB. The retrieval of the LTP in the DG, indication of a functional restoration of the hippocampal neuroplasticity, supported the improvement in cognitive functioning. Interestingly, we observed that, unlike CBDA, CBGA also reduced the depressive‐like behavior, measured as immobility time. The effectiveness of CBGA alone in reducing the depressive‐like behavior suggests that CBGA is able to selectively modulate other molecular targets, possibly of the monoaminergic system (Mendiguren, Aostri, and Pineda 2018), and deserve further investigation. Finally, the recent finding that CBDA and CBGA act as inhibitors of TRPM7 (Suzuki et al. 2023), a receptor channel highly expressed in neuronal cells and supposed to play a relevant role in neurodegenerative diseases (Sun et al. 2015), prompted us to evaluate whether its expression level is affected by the treatment of βA peptide. Indeed, we found that TRPM7 is upregulated in the hippocampus of βA treated mice and such upregulation is reverted to the physiological level by the treatment with both compounds. This result demonstrates that CBDA and CBGA are not only able to target this channel but also modulate its expression level. In this view, we speculate an active role of this receptor channel in contributing to the in vivo efficacy of these compounds. Our results agree with the recent findings of (Kim et al. 2023), who showed that CBDA and THCA are able to rescue memory deficit in Aβ‐treated mice. In summary, we have shown that both CBDA and CBGA are endowed with a multitarget ligand profile, acting not only as dual PPARα/γ agonists but also as inhibitors of both cholinesterase and BACE‐1 enzymes, and molecular targets are currently used in the AD therapy to show down the cognitive impairment associated to the disease, thus providing a rationale for their in vivo activity. However, it should be noted that the limitations of current study, such as the lack of a dose–response curve or the use of only male mice, call for other preclinical and clinical studies to confirm a potential role of CBGA and CBDA in the treatment of neurodegenerative diseases.

## Author Contributions

**Rosa Maria Vitale:** conceptualization, funding acquisition, investigation, methodology, validation, visualization, writing – original draft, writing – review and editing. **Andrea Maria Morace:** formal analysis, investigation. **Antonio D'Errico:** formal analysis, investigation. **Federica Ricciardi:** formal analysis, investigation. **Antimo Fusco:** formal analysis, investigation. **Serena Boccella:** formal analysis, investigation. **Francesca Guida:** methodology, validation, visualization. **Rosarita Nasso:** formal analysis, investigation. **Sebastian Rading:** formal analysis, investigation. **Meliha Karsak:** methodology, supervision, validation. **Diego Caprioglio:** funding acquisition, investigation, resources. **Fabio Arturo Iannotti:** investigation, methodology, resources. **Rosaria Arcone:** methodology, supervision, writing – review and editing. **Livio Luongo:** methodology, validation, visualization. **Mariorosario Masullo:** methodology, supervision, writing – review and editing. **Sabatino Maione:** conceptualization, resources, supervision, writing – review and editing. **Pietro Amodeo:** conceptualization, methodology, writing – original draft, writing – review and editing.

## Conflicts of Interest

In 2017–2018, Rosa Maria Vitale received funding from GW Research Ltd. on Project GWCRI17041.

## Supporting information


Data S1.

## Data Availability

The author have nothing to report.

## References

[ptr8369-bib-0001] Aksoz, E. , B. A. Akyol , and O. Korkut . 2024. “The Role of the Cholinergic System in the Memory‐Protecting Effects of Metformin in a Model of Scopolamine‐Induced Memory Impairment in Aged Rats.” Behavioural Brain Research 466: 114978. 10.1016/j.bbr.2024.114978. 38582410

[ptr8369-bib-0002] Anderson, L. L. , I. K. Low , S. D. Banister , I. S. McGregor , and J. C. Arnold . 2019. “Pharmacokinetics of Phytocannabinoid Acids and Anticonvulsant Effect of Cannabidiolic Acid in a Mouse Model of Dravet Syndrome.” Journal of Natural Products 82, no. 11: 3047–3055. 10.1021/acs.jnatprod.9b00600. 31686510

[ptr8369-bib-0003] Appendino, G. , S. Gibbons , A. Giana , et al. 2008. “Antibacterial Cannabinoids From Cannabis Sativa: A Structure−Activity Study.” Journal of Natural Products 71, no. 8: 1427–1430. 10.1021/np8002673. 18681481

[ptr8369-bib-0004] Atanasov, A. G. , S. B. Zotchev , V. M. Dirsch , and C. T. Supuran . 2021. “Natural Products in Drug Discovery: Advances and Opportunities.” Nature Reviews Drug Discovery 20, no. 3: 200–216. 10.1038/s41573-020-00114-z. 33510482 PMC7841765

[ptr8369-bib-0005] Bajda, M. , A. Więckowska , M. Hebda , N. Guzior , C. Sotriffer , and B. Malawska . 2013. “Structure‐Based Search for New Inhibitors of Cholinesterases.” International Journal of Molecular Sciences 14, no. 3: 5608–5632. 10.3390/ijms14035608. 23478436 PMC3634507

[ptr8369-bib-0006] Barnea, G. , W. Strapps , G. Herrada , et al. 2008. “The Genetic Design of Signaling Cascades to Record Receptor Activation.” Proceedings of the National Academy of Sciences 105, no. 1: 64–69. 10.1073/pnas.0710487105. PMC2224232 18165312

[ptr8369-bib-0007] Case, D. A. , K. Belfon , I. Y. Ben‐Shalom , et al. 2020. “AMBER 2020.” University of California.

[ptr8369-bib-0008] Castelli, V. , G. Lavanco , C. D'Amico , et al. 2023. “CBD Enhances the Cognitive Score of Adolescent Rats Prenatally Exposed to THC and Fine‐Tunes Relevant Effectors of Hippocampal Plasticity.” Frontiers in Pharmacology 14: 1237485. 10.3389/fphar.2023.1237485. 37583903 PMC10424934

[ptr8369-bib-0009] Cheng, C.‐K. , Y.‐C. Tsao , Y.‐C. Su , F.‐C. Sung , H.‐C. Tai , and W.‐M. Kung . 2018. “Metabolic Risk Factors of Alzheimer's Disease, Dementia With Lewy Bodies, and Normal Elderly: A Population‐Based Study.” Behavioural Neurology 2018: 1–8. 10.1155/2018/8312346. PMC6008802 29971140

[ptr8369-bib-0010] Darvesh, S. , M. K. Cash , G. A. Reid , E. Martin , A. Mitnitski , and C. Geula . 2012. “Butyrylcholinesterase Is Associated With β‐Amyloid Plaques in the Transgenic APP SWE /PSEN1dE9 Mouse Model of Alzheimer Disease.” Journal of Neuropathology & Experimental Neurology 71, no. 1: 2–14. 10.1097/NEN.0b013e31823cc7a6. 22157615 PMC3246090

[ptr8369-bib-0048] D’Aniello, F. , T. Fellous , F. A. Iannotti , et al. 2019. “Identification and Characterization of Phytocannabinoids as Novel Dual PPARα/γ Agonists by a Computational and in vitro Experimental Approach.” Biochimica et Biophysica Acta (BBA) ‐ General Subjects 1863, no. 3: 586–597. 10.1016/j.bbagen.2019.01.002. 30611848

[ptr8369-bib-0011] Elahi, F. M. , and B. L. Miller . 2017. “A Clinicopathological Approach to the Diagnosis of Dementia.” Nature Reviews Neurology 13, no. 8: 457–476. 10.1038/nrneurol.2017.96. 28708131 PMC5771416

[ptr8369-bib-0012] Escribano, L. , A.‐M. Simón , E. Gimeno , et al. 2010. “Rosiglitazone Rescues Memory Impairment in Alzheimer's Transgenic Mice: Mechanisms Involving a Reduced Amyloid and Tau Pathology.” Neuropsychopharmacology 35, no. 7: 1593–1604. 10.1038/npp.2010.32. 20336061 PMC3055461

[ptr8369-bib-0013] Esposito, G. , C. Scuderi , C. Savani , et al. 2007. “Cannabidiol In Vivo Blunts β‐Amyloid Induced Neuroinflammation by Suppressing IL‐1β and iNOS Expression.” British Journal of Pharmacology 151, no. 8: 1272–1279. 10.1038/sj.bjp.0707337. 17592514 PMC2189818

[ptr8369-bib-0014] Esposito, G. , C. Scuderi , M. Valenza , et al. 2011. “Cannabidiol Reduces Aβ‐Induced Neuroinflammation and Promotes Hippocampal Neurogenesis Through PPARγ Involvement.” PLoS One 6, no. 12: e28668. 10.1371/journal.pone.0028668. 22163051 PMC3230631

[ptr8369-bib-0015] Ferreira, J. P. S. , H. M. T. Albuquerque , S. M. Cardoso , A. M. S. Silva , and V. L. M. Silva . 2021. “Dual‐Target Compounds for Alzheimer's Disease: Natural and Synthetic AChE and BACE‐1 Dual‐Inhibitors and Their Structure‐Activity Relationship (SAR).” European Journal of Medicinal Chemistry 221: 113492. 10.1016/j.ejmech.2021.113492. 33984802

[ptr8369-bib-0016] Fox, T. , and P. A. Kollman . 1998. “Application of the RESP Methodology in the Parametrization of Organic Solvents.” Journal of Physical Chemistry B 102, no. 41: 8070–8079. 10.1021/jp9717655.

[ptr8369-bib-0017] Govindarajulu, M. , P. D. Pinky , J. Bloemer , N. Ghanei , V. Suppiramaniam , and R. Amin . 2018. “Signaling Mechanisms of Selective PPAR γ Modulators in Alzheimer's Disease.” PPAR Research 2018: 1–20. 10.1155/2018/2010675. PMC6215547 30420872

[ptr8369-bib-0018] Guida, F. , M. Iannotta , G. Misso , et al. 2022. “Long‐Term Neuropathic Pain Behaviors Correlate With Synaptic Plasticity and Limbic Circuit Alteration: A Comparative Observational Study in Mice.” Pain 163, no. 8: 1590–1602. 10.1097/j.pain.0000000000002549. 34862336 PMC9341227

[ptr8369-bib-0019] Iannotti, F. A. , F. De Maio , E. Panza , et al. 2020. “Identification and Characterization of Cannabimovone, a Cannabinoid From Cannabis Sativa, as a Novel PPARγ Agonist via a Combined Computational and Functional Study.” Molecules 25, no. 5: 1119. 10.3390/molecules25051119. 32138197 PMC7179127

[ptr8369-bib-0049] Infantino, R. , S. Boccella , D. Scuteri , et al. 2022. “2‐Pentadecyl‐2‐Oxazoline Prevents Cognitive and Social Behaviour Impairments in the Amyloid β‐Induced Alzheimer‐like Mice Model: Bring the α2 Adrenergic Receptor back into Play.” Biomed Pharmacother 156: 113844. 10.1016/j.biopha.2022.113844. 36252359

[ptr8369-bib-0020] Karl, T. , B. Garner , and D. Cheng . 2017. “The Therapeutic Potential of the Phytocannabinoid Cannabidiol for Alzheimer's Disease.” Behavioural Pharmacology 28, no. 2 and 3: 142–160. 10.1097/FBP.0000000000000247. 27471947

[ptr8369-bib-0021] Kim, H. Y. , D. K. Lee , B.‐R. Chung , H. V. Kim , and Y. Kim . 2016. “Intracerebroventricular Injection of Amyloid‐β Peptides in Normal Mice to Acutely Induce Alzheimer‐like Cognitive Deficits.” Journal of Visualized Experiments 109: 53308. 10.3791/53308. PMC4829024 27023127

[ptr8369-bib-0022] Kim, J. , P. Choi , Y.‐T. Park , T. Kim , J. Ham , and J.‐C. Kim . 2023. “The Cannabinoids, CBDA and THCA, Rescue Memory Deficits and Reduce Amyloid‐Beta and tau Pathology in an Alzheimer's Disease‐Like Mouse Model.” International Journal of Molecular Sciences 24, no. 7: 6827. 10.3390/ijms24076827. 37047798 PMC10095267

[ptr8369-bib-0023] Koch, G. , and D. Spampinato . 2022. “Alzheimer Disease and Neuroplasticity.” Handbook of Clinical Neurology 184: 473–479. 10.1016/B978-0-12-819410-2.00027-8. 35034755

[ptr8369-bib-0024] Kroeze, W. K. , M. F. Sassano , X.‐P. Huang , et al. 2015. “PRESTO‐Tango as an Open‐Source Resource for Interrogation of the Druggable Human GPCRome.” Nature Structural & Molecular Biology 22, no. 5: 362–369. 10.1038/nsmb.3014. PMC4424118 25895059

[ptr8369-bib-0025] Legare, C. A. , W. M. Raup‐Konsavage , and K. E. Vrana . 2022. “Therapeutic Potential of Cannabis, Cannabidiol, and Cannabinoid‐Based Pharmaceuticals.” Pharmacology 107, no. 3–4: 131–149. 10.1159/000521683. 35093949

[ptr8369-bib-0026] Livingston, G. , A. Sommerlad , V. Orgeta , et al. 2017. “Dementia Prevention, Intervention, and Care.” Lancet 390, no. 10113: 2673–2734. 10.1016/S0140-6736(17)31363-6. 28735855

[ptr8369-bib-0027] Maiuolo, J. , P. Costanzo , M. Masullo , et al. 2023. “Hydroxytyrosol–Donepezil Hybrids Play a Protective Role in an In Vitro Induced Alzheimer's Disease Model and in Neuronal Differentiated Human SH‐SY5Y Neuroblastoma Cells.” International Journal of Molecular Sciences 24, no. 17: 13461. 10.3390/ijms241713461. 37686262 PMC10488223

[ptr8369-bib-0028] Mendiguren, A. , E. Aostri , and J. Pineda . 2018. “Regulation of Noradrenergic and Serotonergic Systems by Cannabinoids: Relevance to Cannabinoid‐Induced Effects.” Life Sciences 192: 115–127. 10.1016/j.lfs.2017.11.029. 29169951

[ptr8369-bib-0029] Mhillaj, E. , M. G. Morgese , P. Tucci , et al. 2018. “Celecoxib Prevents Cognitive Impairment and Neuroinflammation in Soluble Amyloid β‐Treated Rats.” Neuroscience 372: 58–73. 10.1016/j.neuroscience.2017.12.046. 29306052

[ptr8369-bib-0030] Moore, C. F. , J. Marusich , M. Haghdoost , T. W. Lefever , M. O. Bonn‐Miller , and E. M. Weerts . 2023. “Evaluation of the Modulatory Effects of Minor Cannabinoids and Terpenes on Delta‐9‐Tetrahydrocannabinol Discrimination in Rats.” Cannabis and Cannabinoid Research 8, no. S1: S42–S50. 10.1089/can.2023.0062. 37721992

[ptr8369-bib-0031] Morris, G. M. , R. Huey , W. Lindstrom , et al. 2009. “AutoDock4 and AutoDockTools4: Automated Docking With Selective Receptor Flexibility.” Journal of Computational Chemistry 30, no. 16: 2785–2791. 10.1002/jcc.21256. 19399780 PMC2760638

[ptr8369-bib-0032] Mushtaq, G. , N. Greig , J. Khan , and M. Kamal . 2014. “Status of Acetylcholinesterase and Butyrylcholinesterase in Alzheimer's Disease and Type 2 Diabetes Mellitus.” CNS & Neurological Disorders–Drug Targets 13, no. 8: 1432–1439. 10.2174/1871527313666141023141545. 25345511 PMC5878042

[ptr8369-bib-0033] Pettersen, E. F. , T. D. Goddard , C. C. Huang , et al. 2004. “UCSF Chimera—A Visualization System for Exploratory Research and Analysis.” Journal of Computational Chemistry 25, no. 13: 1605–1612. 10.1002/jcc.20084. 15264254

[ptr8369-bib-0034] Prati, F. , G. Bottegoni , M. L. Bolognesi , and A. Cavalli . 2018. “BACE‐1 Inhibitors: From Recent Single‐Target Molecules to Multitarget Compounds for Alzheimer's Disease.” Journal of Medicinal Chemistry 61, no. 3: 619–637. 10.1021/acs.jmedchem.7b00393. 28749667

[ptr8369-bib-0035] Rathod, S. S. , and Y. O. Agrawal . 2024. “Phytocannabinoids as Potential Multitargeting Neuroprotectants in Alzheimer's Disease.” Current Drug Research Reviews 16, no. 2: 94–110. 10.2174/2589977515666230502104021. 37132109

[ptr8369-bib-0036] Reid, G. A. , and S. Darvesh . 2015. “Butyrylcholinesterase‐Knockout Reduces Brain Deposition of Fibrillar β‐Amyloid in an Alzheimer Mouse Model.” Neuroscience 298: 424–435. 10.1016/j.neuroscience.2015.04.039. 25931333

[ptr8369-bib-0037] Rossi, M. , M. Freschi , L. de Camargo Nascente , et al. 2021. “Sustainable Drug Discovery of Multi‐Target‐Directed Ligands for Alzheimer's Disease.” Journal of Medicinal Chemistry 64, no. 8: 4972–4990. 10.1021/acs.jmedchem.1c00048. 33829779 PMC8154578

[ptr8369-bib-0038] Sato, T. , H. Hanyu , K. Hirao , H. Kanetaka , H. Sakurai , and T. Iwamoto . 2011. “Efficacy of PPAR‐γ Agonist Pioglitazone in Mild Alzheimer Disease.” Neurobiology of Aging 32, no. 9: 1626–1633. 10.1016/j.neurobiolaging.2009.10.009. 19923038

[ptr8369-bib-0039] Schmidt, M. W. , K. K. Baldridge , J. A. Boatz , et al. 1993. “General Atomic and Molecular Electronic Structure System.” Journal of Computational Chemistry 14, no. 11: 1347–1363. 10.1002/jcc.540141112.

[ptr8369-bib-0040] Sun, Y. , P. Sukumaran , A. Schaar , and B. B. Singh . 2015. “TRPM7 and Its Role in Neurodegenerative Diseases.” Channels 9, no. 5: 253–261. 10.1080/19336950.2015.1075675. 26218331 PMC4826135

[ptr8369-bib-0041] Suzuki, S. , C. Wakano , M. K. Monteilh‐Zoller , A. J. Cullen , A. Fleig , and R. Penner . 2023. “Cannabigerolic Acid (CBGA) Inhibits the TRPM7 Ion Channel Through Its Kinase Domain.” Function 5, no. 1: zqad069. 10.1093/function/zqad069. 38162115 PMC10757070

[ptr8369-bib-0042] Taing, K. , L. Chen , and H.‐R. Weng . 2023. “Emerging Roles of GPR109A in Regulation of Neuroinflammation in Neurological Diseases and Pain.” Neural Regeneration Research 18, no. 4: 763–768. 10.4103/1673-5374.354514. 36204834 PMC9700108

[ptr8369-bib-0043] Tommonaro, G. , N. García‐Font , R. M. Vitale , et al. 2016. “Avarol Derivatives as Competitive AChE Inhibitors, Non Hepatotoxic and Neuroprotective Agents for Alzheimer's Disease.” European Journal of Medicinal Chemistry 122: 326–338. 10.1016/j.ejmech.2016.06.036. 27376495

[ptr8369-bib-0044] Tucci, P. , E. Mhillaj , M. G. Morgese , et al. 2014. “Memantine Prevents Memory Consolidation Failure Induced by Soluble Beta Amyloid in Rats.” Frontiers in Behavioral Neuroscience 8: 332. 10.3389/fnbeh.2014.00332. 25285073 PMC4168698

[ptr8369-bib-0045] Vitale, R. M. , F. A. Iannotti , and P. Amodeo . 2021. “The (Poly)pharmacology of Cannabidiol in Neurological and Neuropsychiatric Disorders: Molecular Mechanisms and Targets.” International Journal of Molecular Sciences 22, no. 9: 4876. 10.3390/ijms22094876. 34062987 PMC8124847

[ptr8369-bib-0046] Vitale, R. M. , V. Rispoli , D. Desiderio , et al. 2018. “In Silico Identification and Experimental Validation of Novel Anti‐Alzheimer's Multitargeted Ligands From a Marine Source Featuring a “2‐Aminoimidazole Plus Aromatic Group” Scaffold.” ACS Chemical Neuroscience 9, no. 6: 1290–1303. 10.1021/acschemneuro.7b00416. 29473731

[ptr8369-bib-0047] Wójtowicz, S. , A. K. Strosznajder , M. Jeżyna , and J. B. Strosznajder . 2020. “The Novel Role of PPAR Alpha in the Brain: Promising Target in Therapy of Alzheimer's Disease and Other Neurodegenerative Disorders.” Neurochemical Research 45, no. 5: 972–988. 10.1007/s11064-020-02993-5. 32170673 PMC7162839

