# Supplementary material for: Identification of Cannabidiolic and Cannabigerolic Acids as MTDL AChE, BuChE, and BACE‐1 Inhibitors Against Alzheimer's Disease by In Silico, In Vitro, and In Vivo Studies
Source: Phytother Res. 2024 Nov 7;39(1):233–45. doi: 10.1002/ptr.8369 (PMC11745148; doi:10.1002/ptr.8369)
Supplement: Supplementary file 1 — Data S1. [file PTR-39-233-s001.docx]

**Supplementary material**

**The non-psychoactive phytocannabinoids cannabidiolic and cannabigerolic acids as multitarget ligands against Alzheimer’s disease**

**Rosa Maria Vitale^1^, Andrea Maria Morace^2^, Antonio D’Errico^3^, Federica Ricciardi^2^, Serena Boccella^2^, Francesca Guida^2^, Rosarita Nasso^3^, Sebastian Rading^4,5^, Meliha Karsak^4,5^, Diego Caprioglio^6^, Fabio Arturo Iannotti^1^, Rosaria Arcone^3^, Livio Luongo^2^, Mariorosario Masullo^3^, Sabatino Maione^2^ and Pietro Amodeo^1^**

**1 Institute of Biomolecular Chemistry (ICB)- National Research Council (CNR)- Via Campi Flegrei 34, 80078 Pozzuoli (NA)-Italy**

**2 Department of Experimental Medicine, Division of Pharmacology, University of Campania "Luigi Vanvitelli", Via S.M. di Costantinopoli, 16, 80138, Naples, Italy**

**3 Department of Medical, Health and Well-being Sciences, University of Naples “Parthenope”, Via Medina 40, 80133 Naples, Italy**

**4 Neuronal and Cellular Signal Transduction, Center for Molecular Neurobiology Hamburg (ZMNH), University Medical Center Hamburg-Eppendorf (UKE), 20251 Hamburg, Germany**

**5 Institute of Human Genetics, University Medical Center Hamburg-Eppendorf (UKE), 20251 Hamburg, Germany**

**6 Department of Pharmaceutical and Pharmacological Sciences, University of Eastern Piedmont "A. Avogadro", Via Bovio 6, 28100 Novara (Italy)**

1. **H^1^ NMR spectra**

^1^H 400 MHz NMR spectra were measured on Bruker 400 spectrometers (Bruker®, Billerica, MA, USA). Chemical shifts were referenced to the residual solvent signal (CDCl_3_: δ_H_ = 7.26)


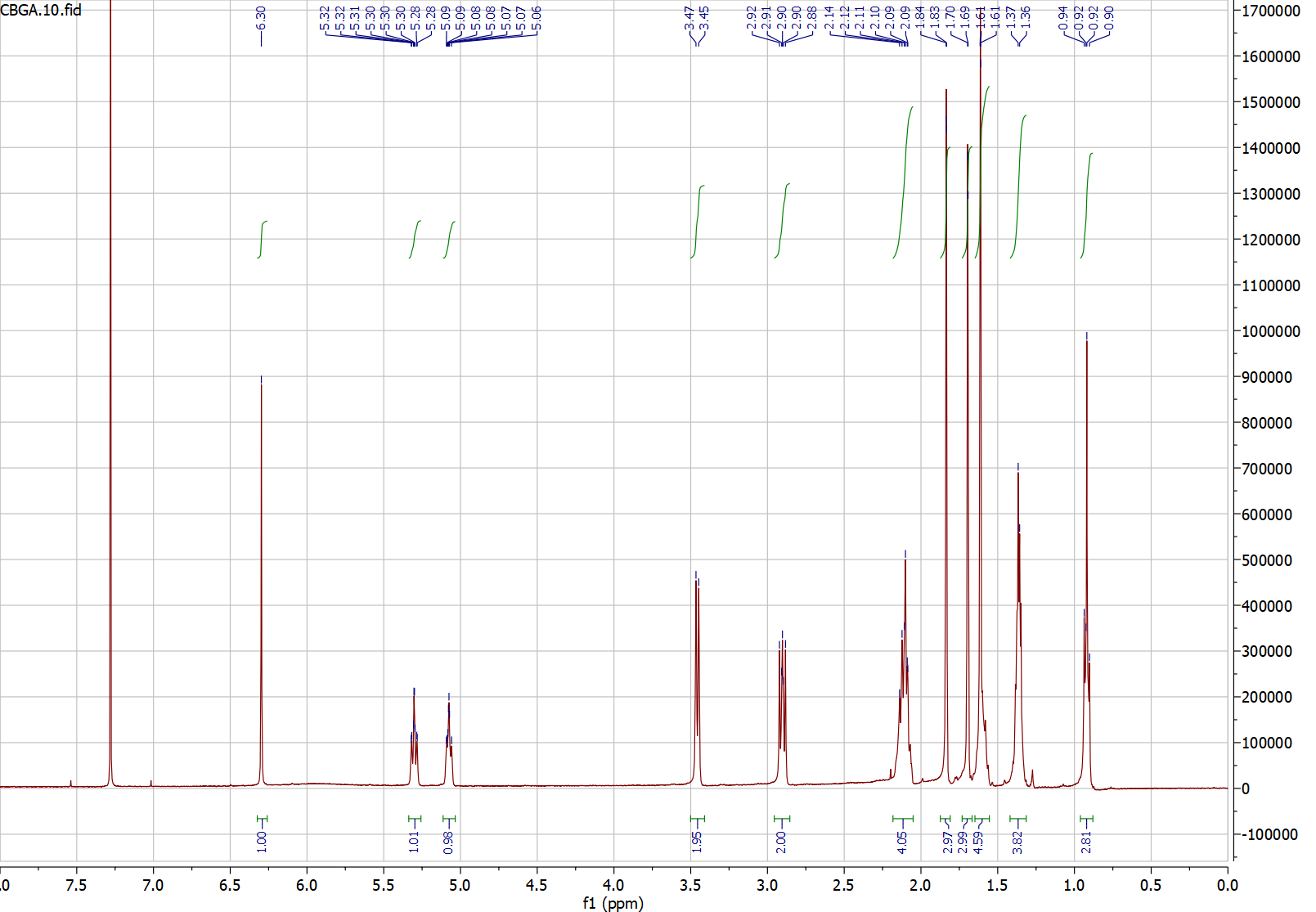


***Figure S1.*** *^1^H NMR of CBGA*

***
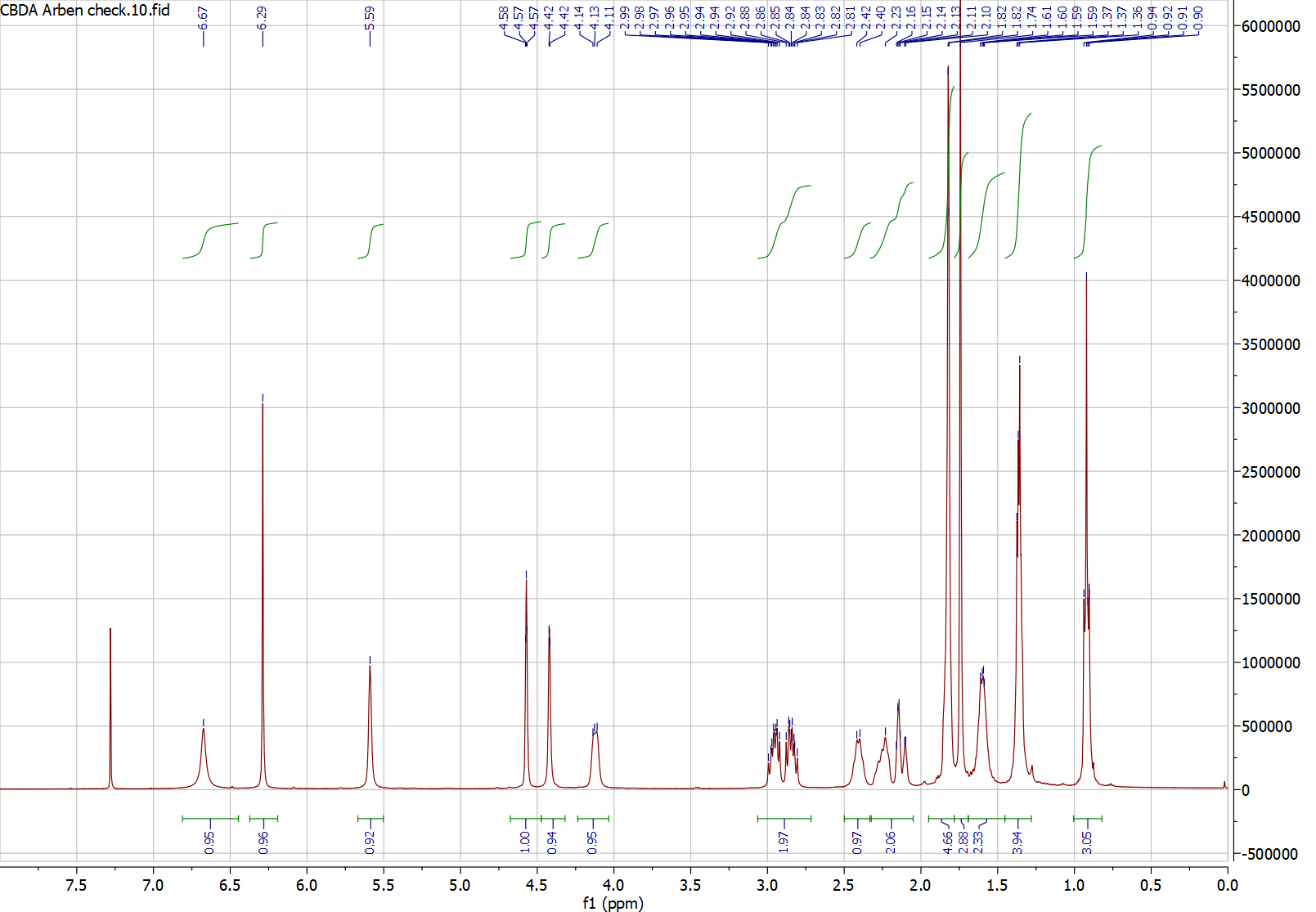
Figure S2*** *. ^1^H NMR of CBDA*

1. **
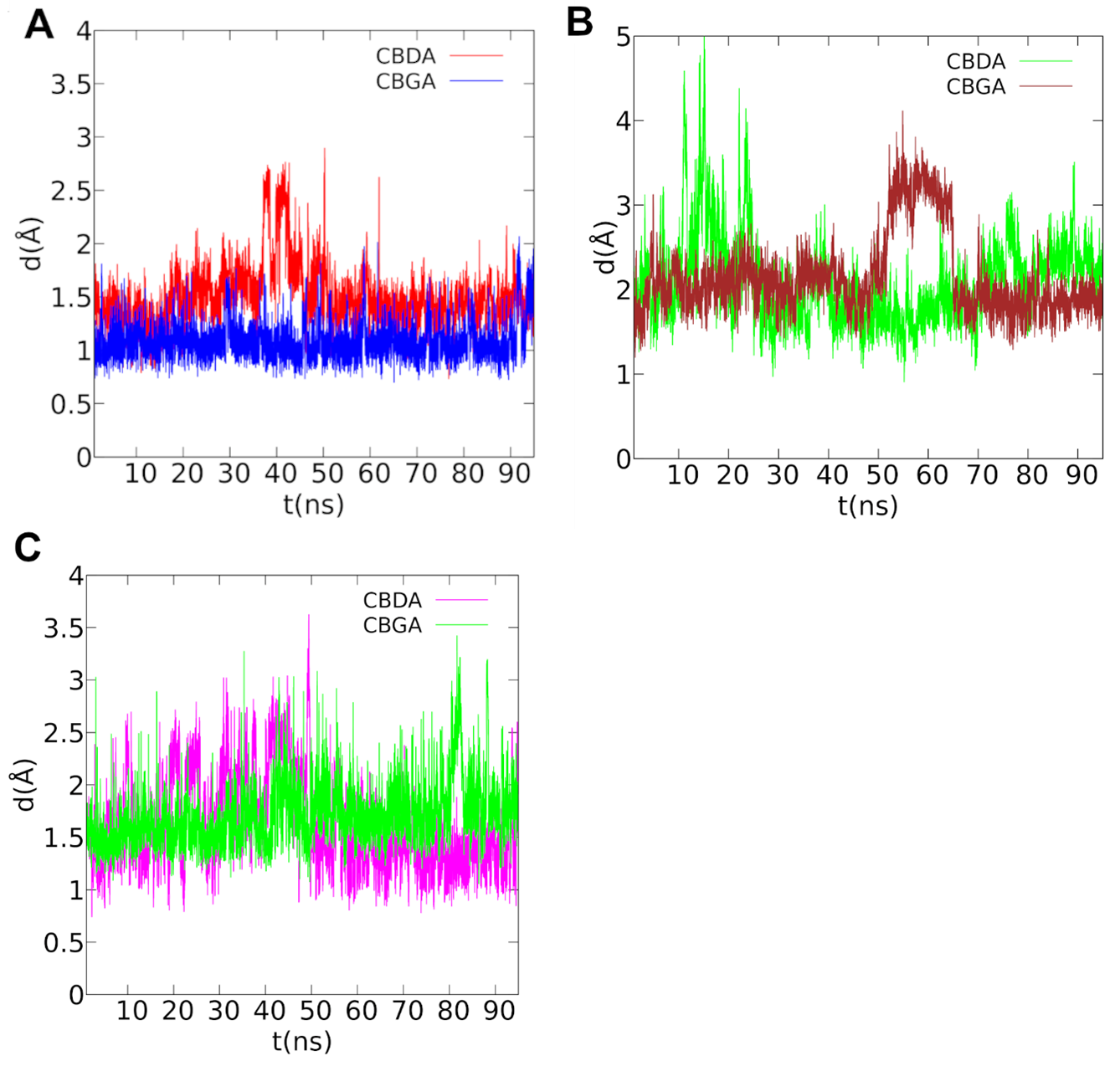
Molecular dynamics rmsd plots for AChE, BuChE and BACE-1 in complex with CBGA and CBDA *Figure S3.*** *Root-mean-square deviation (RMSD) plots of CBDA and CBGA in complex with AChE (A), BuChE (B) and BACE-1 (C) over last 90ns of MD trajectories after best fitting of protein backbones. The ligand RMSD plot was smoothed with a five-point window running average.*
2. **Determination of Cholinesterase activity kinetic parameters**

Cholinesterase activity was assayed by the Elmann method using acetylthiocholine or butyrylthiocholine as substrate for AChE or BuChE, respectively. In particular, the reduction of DTNB was followed colorimetrically (412 nm) at room temperature (22-27°C). The reaction mixture (500 µL) contained 330 µM DTNB in 0.1 M sodium phosphate buffer, pH 7.1 and the reaction was started by the addition of 100 mU/ml AChE or BuChE, respectively, and the initial rate of the reaction was derived from the linear portion of the kinetics, determined at different substrate concentration. The kinetic parameters of the enzymatic reaction *K*_m_ and *V*_max_ were derived from the determination of the enzyme activity at different substrate concentration (50 – 500 µM). The data were either interpolated in the hyperbolic Michaelis-Menten equation or linearized with the Lineweaver-Burk equation, giving similar results.


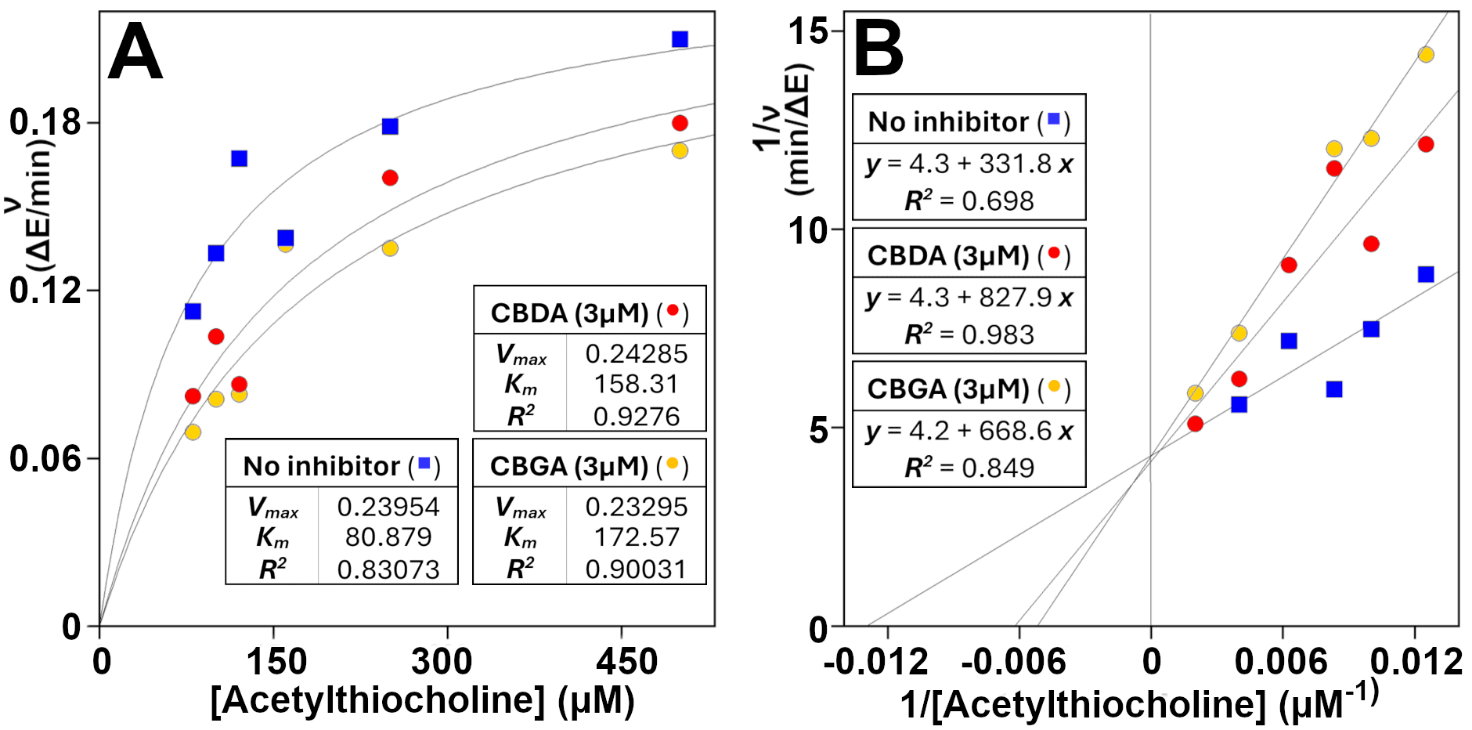


***Figure S4. Representative experiment of the effect of CBDA and CBGA on the kinetics of AChE. Panel A****. The initial rate of acethylthiocholine hydrolysis by AChE was measured at the indicated concentration and non-linear interpolated in hyperbolic Michaelis-Menten equation.* ***Panel B****. Lineveawer-Burk transformation of the data reported in Panel A. Insets: correlation data.*


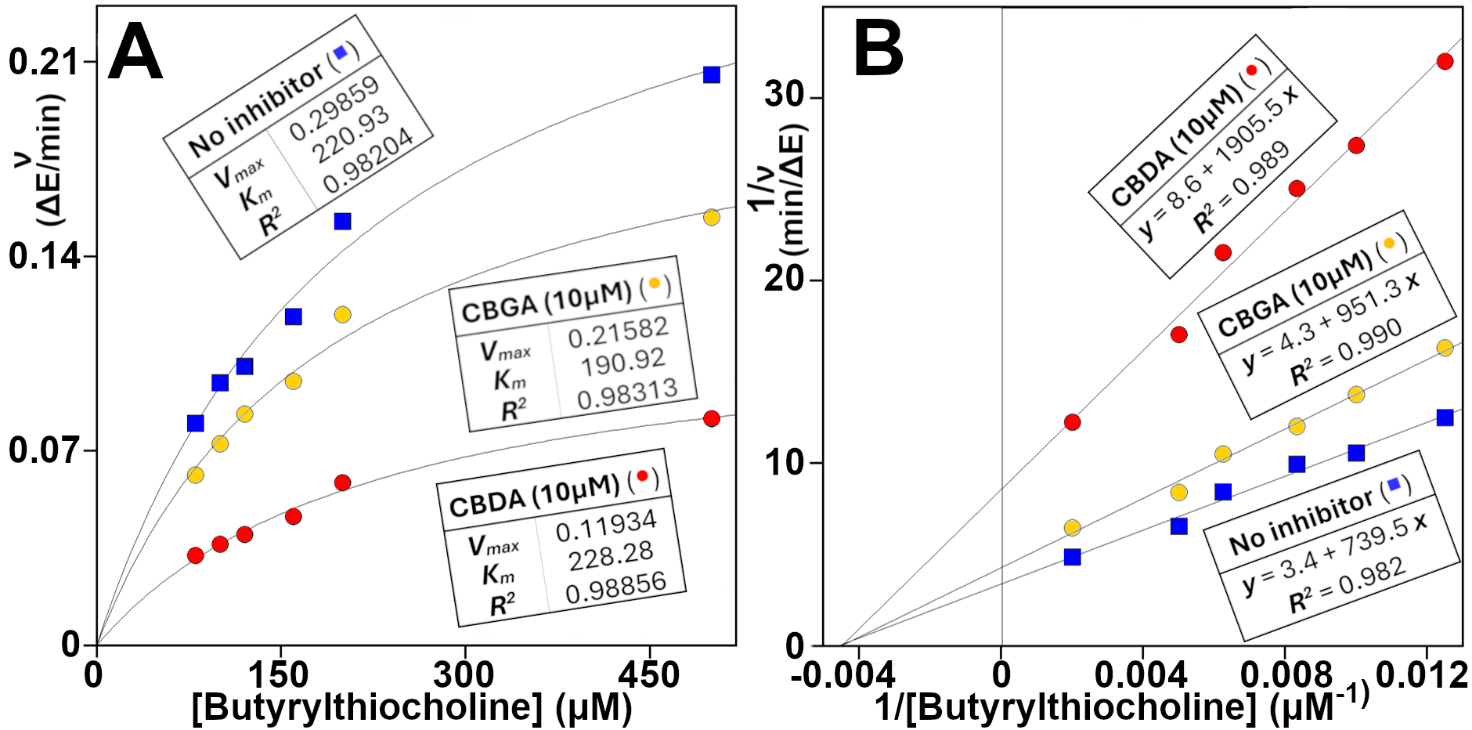


***Figure S5. Representative experiment of the effect of CBDA and CBGA on the kinetics of BuChE. Panel A****. The initial rate of butirrylthiocholine hydrolysis by BuChE was measured at the indicated concentration and non-linear interpolated in hyperbolic Michaelis-Menten equation.* ***Panel B****. Lineveawer-Burk transformation of the data reported in Panel A. Insets: correlation data.*

1. **Inhibition constants determination**

The values of *K*_i_ were derived from the equations, *K*_m_' = *K*_m_ (1 + [I]/*K*_i_) in the case of competitive inhibition, and *V*_max_' = *V*_max_ (1 + [I]/*K*_i_), in the case of non-competitive inhibition. *V*_max_' and *K*_m_' represent the value of *V*_max_ and *K*_m_ measured at the [I] concentration of inhibitor, respectively.

***Table S1. Inhibition parameters of CBDA and CBGA on AChE***

*Values of K_i_ were derived only from the increase of K_m_ (competitive inhibition).*

| Compound | µM | *V*_max_ | | *K*_m_ | | *K*_i_ | |
| --- | --- | --- | --- | --- | --- | --- | --- |
|  |  | LB* | MM** | LB* | MM** | LB* | MM** |
| -- | 0 | 0.192±0.007 | 0.190±0.009 | 131.9±2.3 | 125.9±5.1 | - | - |
| CBDA | 1.25 | 0.211 | 0.208 | 134.9 | 130.8 | 55.0*** | 32.1*** |
| “ | 1.75 | 0.242 | 0.212 | 187.3 | 141.8 | 4.2 | 13.9 |
| “ | 2.50 | 0.233 | 0.255 | 163.3 | 190.8 | 10.5 | 4.8 |
| “ | 3.00 | 0.248 | 0.301 | 169.2 | 237.5 | 10.6 | 3.4 |
| CBGA | 1.25 | 0.196 | 0.196 | 144.1 | 143.1 | 13.5 | 9.1 |
| “ | 2.50 | 0.201 | 0.190 | 166.1 | 151.8 | 9.6 | 12.2 |
| “ | 3.00 | 0.232 | 0.233 | 192.4 | 172.6 | 6.5 | 8.1 |
| “ | 3.75 | 0.209 | 0.196 | 166.3 | 143.6 | 14.4 | 26.7*** |

* Lineweaver-Burk equation

** Michaelis-Menten interpolation

*** Not included in the mean calculation of values reported in the manuscript.

***Table S2. Inhibition parameters of CBDA and CBGA on BuChE***

*Values of K_i_ were derived only from the decrease of V_max_ (non-competitive inhibition).*

| Compound | Exp. | µM | *V*_max_ | | *K*_m_ | | *K*_i_ | |
| --- | --- | --- | --- | --- | --- | --- | --- | --- |
|  |  |  | LB* | MM** | LB* | MM** | LB* | MM** |
| -- | 1 | 0 | 0.295 | 0.299 | 218.5 | 220.9 | - | - |
| CBDA | 1 | 10 | 0.112 | 0.119 | 204.5 | 228.3 | 6.1 | 6.6 |
| CBGA | 1 | 10 | 0.235 | 0.216 | 223.1 | 190.9 | 39.2 | 26.0 |
| -- | 2 | 0 | 0.220 | 0.249 | 195.0 | 235.8 | - | - |
| CBDA | 2 | 2.66 | 0.162 | 0.0.176 | 182.0 | 209.9 | 7.5 | 6.4 |
| GA | 2 | 2.66 | 0.185 | 0.209 | 158.3 | 196.9 | 14.1 | 14.0 |

* Lineweaver-Burk equation

** Michaelis-Menten interpolation

1. **Beta-arrestin assay on GPR109A**

***Figure S6.*** *CBDA and CBGA do not activate GPR109A receptor by β-arrestin recruitment. Similar relative luminescence units (RLU) were determined in the β-arrestin recruitment assay for HCA2 receptor stimulation with (A) CBDA and (B) CBGA in comparison to vehicle treatment. In contrast, the cannabinoid CB2 receptor (CNR2-Tango) with its agonist JWH-133 (positive control) produced a strong β-arrestin recruitment in this reporter-gene based assay. Mean values +/- SD are given for nine replicate wells (for CB2 two replicates). Data were analyzed by a nonparametric one-way ANOVA (Kruskal-Wallis test) or t-test, with statistical significance set at P < 0.05. Data analysis and graphs were generated using GraphPad Prism Software.*
